# Clinical Effectiveness of Bulk-Fill and Conventional Resin Composite Restorations: Systematic Review and Meta-Analysis

**DOI:** 10.3390/polym12081786

**Published:** 2020-08-10

**Authors:** Heber Isac Arbildo-Vega, Barbara Lapinska, Saurav Panda, César Lamas-Lara, Abdul Samad Khan, Monika Lukomska-Szymanska

**Affiliations:** 1 Department of General Dentistry, Dentistry School, Universidad San Martín de Porres, Chiclayo 14012, Peru; harbildov@usmp.pe; 2 Department of General Dentistry, Dentistry School, Universidad Particular de Chiclayo, Chiclayo 14012, Peru; 3 Department of General Dentistry, Medical University of Lodz, 92-213 Lodz, Poland; barbara.lapinska@umed.lodz.pl; 4 Department of Periodontics and Oral Implantology, Siksha ‘O’ Anusandhan Univeristy, Bhubaneswar 751003, India; sauravpanda@soa.ac.in; 5 Department of Biomedical, Surgical and Dental Sciences, Universita Degli Studi di Milano, 20122 Milano, Italy; 6 Department of General Dentistry, Stomatology School, Universidad Peruana Los Andes, Lima 15072, Peru; d.clamas@upla.edu.pe; 7 Department of Restorative Dental Sciences, College of Dentistry, Imam Abdulrahman Bin Faisal University, Dammam 31441, Saudi Arabia; akhan@iau.edu.sa

**Keywords:** bulk-fill resin, resin composite, dental restoration, systematic review, meta-analysis

## Abstract

The objective of this systematic review and meta-analysis was to determine the clinical effectiveness of bulk-fill and conventional resin in composite restorations. A bibliographic search was carried out until May 2020, in the biomedical databases Pubmed/MEDLINE, EMBASE, Scopus, CENTRAL and Web of Science. The study selection criteria were: randomized clinical trials, in English, with no time limit, with a follow-up greater than or equal to 6 months and that reported the clinical effects (absence of fractures, absence of discoloration or marginal staining, adequate adaptation marginal, absence of post-operative sensitivity, absence of secondary caries, adequate color stability and translucency, proper surface texture, proper anatomical form, adequate tooth integrity without wear, adequate restoration integrity, proper occlusion, absence of inflammation and adequate point of contact) of restorations made with conventional and bulk resins. The risk of bias of the study was analyzed using the Cochrane Manual of Systematic Reviews of Interventions. Sixteen articles were eligible and included in the study. The results indicated that there is no difference between restorations with conventional and bulk resins for the type of restoration, type of tooth restored and restoration technique used. However, further properly designed clinical studies are required in order to reach a better conclusion.

## 1. Introduction

Currently, the most common dental problem is dental caries, which is characterized as a bacterial infection causing damage to the tooth structure. For its treatment, dentists recommend removing carious dental tissue and filling the resulting cavity with the appropriate restorative materials. Currently, polymeric compounds are used as dental restorative materials due to their good physical, mechanical, thermal, and tribological properties [1]. The increasing demand for aesthetic, tooth-colored, and mercury-free restoration has driven a surge in the use of resin-based composite dental materials [2]. Direct dental restorations should withstand occlusal loading, minimize or prevent stress development and avoid gap formation, be stable in oral environments, and be easy to use. Preferably, these restorations should also prevent biofilm attachment, present remineralization capabilities, and be able to self-repair. To date, no commercially available material is able to meet all of these requirements [3], however attempts are being made to develop new resin materials containing bisphenol A-free monomers [4]. According to the guidelines of the Academy of Dental Materials [5], the highest priorities for evaluating resin composites are strength, elastic modulus, fracture toughness, fatigue, indentation hardness, and wear (abrasion and attrition) measurements. Next, toughness, edge strength (chipping), and wear determined by toothbrush should be evaluated.

In the last decade, composite resin restorations have evolved exponentially and considerably in terms of both their optical (better aesthetics) and mechanical properties. However, some of the limitations are resistance to fracture, volumetric contraction that results from the polymerization of the material, and the development of polymerization stress [5,6].

An incremental technique for composite resin placement was developed to overcome polymerization shrinkage of microhybrid composites. However, this technique is time-consuming and may lead to air entrapment between consecutive layers of the composite resin. In order to reduce the undesired effects of the composites, such as the tension created on the tooth or restoration interface, some chemical and structural changes in the composite resin composition have been proposed. These include modifications in the resin matrix, quantity, shape, or surface treatment of the inorganic particle [7]. Currently, bulk-fill resin composites are the materials of choice in direct dental restorations. They possess lower post-gel shrinkage and higher reactivity to light polymerization than most conventional composites as a result of their increased translucency, improving the light penetration and the depth of cure [8,9]. The abovementioned features allow for placement of 4–5-mm-thick increments of bulk-fill material, shortening the clinical procedure and facilitating handling. Due to their different clinical uses, bulk-fill composites can be categorized as either base or full-body bulk-fill resin composites [9]. Base bulk-fill composites have low viscosity, allowing for their placement and adaptation in deep cavities. However, their lower filler content, which results in lower wear resistance, requires the base of the bulk-fill to be covered with a conventional composite (two-step bulk technique). Full-body bulk-fill composites, however, have a higher filler load, making them highly viscous and resistant to wear. As such, these paste-like bulk-fill materials can be placed in the cavity without any coverage (bulk technique) [10].

Bulk-fill composites were reported to promote less polymerization shrinkage stress than conventional microhybrid composite during and after the light curing process in class II posterior resin composite restorations [11].

However, since the introduction of bulk-fill composite resins on the market, many studies have been conducted comparing the different properties between conventional resins and bulk-fill resins, reporting conflicting results [11]. Bulk-fill composites are a tempting alternative due to their fast and easy application protocol, while conventional composites are thought to possess well-documented clinical performance. Thus, clinical dentists are still unsure about the adoption of this new class of materials in clinical practice.

Therefore, the aim of this systematic review and meta-analysis was to evaluate the clinical performance of bulk-fill resin composites used in direct restorations and compare them with conventional resin composites. The null hypothesis of the study was that the clinical effectiveness of these resin composites is comparable.

## 2. Materials and Methods

This review was carried out following the Preferred Reporting Items for Systematic Reviews and Meta-Analyses (PRISMA) statement guidelines [12].

### 2.1. Search Strategy

CENTRAL (Cochrane Central Controlled Trials Register), EMBASE (Excerpta Medica database), MEDLINE (Bibliographic reference base of the U.S National Library of Medicine)/PubMed, Scopus, and Web of Science were searched for clinical trial findings. The initial review carried out with the structured protocol was adopted for the search, the following keywords of which were: (“dental caries” or “dental restoration, permanent”) AND (“bulk fill” or “bulkfill” or “bulk-fill” or “bulk”) AND (“composite resins” or “composite resin” or “resin composite” or “resin composites” or “resin restoration” or “composite restoration” or “composite restorations”). The search of the literature was performed without any date limits and was done up until May 2020.

### 2.2. Study Selection

Full texts of papers were obtained from the journals. The inclusion and exclusion criteria for articles are presented in Table 1.

### 2.3. Study Quality Assessment

The title and abstracts of all the articles identified by the electronic search were read and evaluated by four authors (M.L.-S., B.L., A.S.K., and S.P.) Disagreements between the reviewers were resolved by consensus with all the authors.

To evaluate the studies, a duplicate checklist was performed to extract the information of interest and change the data. Three reviewers (ML-S., A.S.K., and B.L.) independently assessed articles by name, author, year of publication, type of study, number of patients (male-female ratio), number of teeth restored, mean age and age range of patients, follow-up time, country where the study was conducted, study groups, number of patients and teeth per study group, type of restoration (class I, II, or V), type of tooth (incisor, canine, premolar, and molar), evaluation criteria, etching method, adhesive used, resin used, techniques used (incremental, bulk or bulk two-step), and the clinical parameters evaluated by each study. To resolve any discrepancies between the reviewers, they were discussed together with a third reviewer (H.I.A.-V.) to reach an agreement.

### 2.4. Assessment of the Risk of Bias of the Studies

The risk of bias assessment of each study was carried out by three authors (H.I.A.-V., S.P., and C.L.-L.) and was analyzed according to the Cochrane Handbook of Systematic Reviews of Interventions [13]. For the risk of bias, 7 items were analyzed and articles were grouped as being high, moderate, or low risk. An article was considered as low risk of bias, if all your items met the standards of the Cochrane Handbook of Systematic Reviews of Interventions; moderate risk of bias, if 1 or more items were doubtful; and high risk of bias, if 2 or more items did not comply with the regulations of the Cochrane Handbook of Systematic Reviews of Interventions [13].

### 2.5. Analysis of Results

The data from each study were placed and analyzed in the RevMan 5.3 program (Cochrane Group, London, UK) using a relative risk (RR) measure and with a 95% confidence interval (CI).

## 3. Results

### 3.1. Selection of Studies

**Figure 1:**
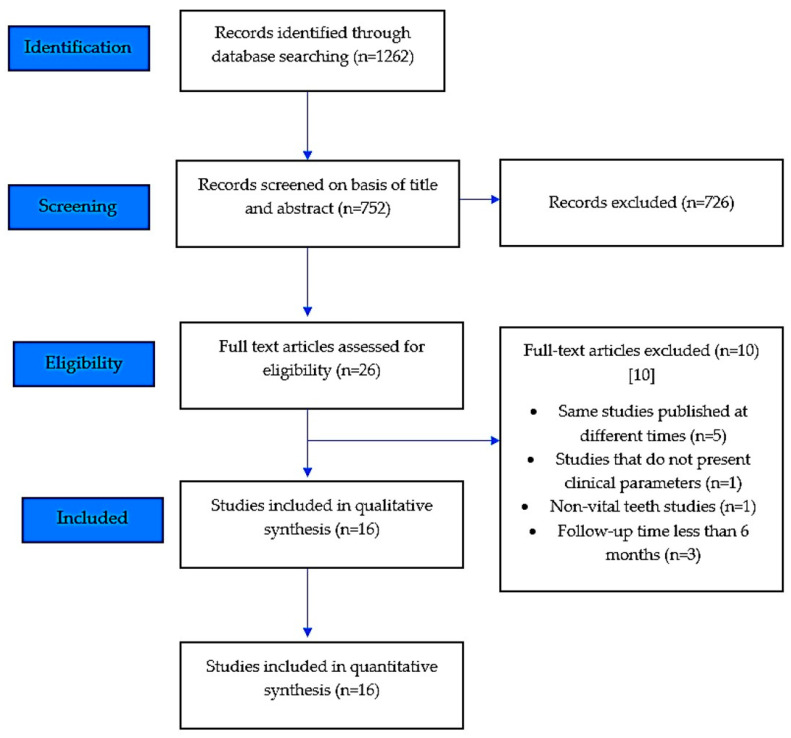
PRISMA flow diagram of the literature search and selection process. Note: PRISMA = Preferred Reporting Items for Systematic Reviews and Meta-Analyses [12].

A total of 1262 titles were identified from the database search carried out until May 2020. After removing the duplicates, 752 titles were thoroughly assessed based on the selection criteria. The full-text assessment was carried out for 26 potentially eligible studies to identify 16 titles meeting the inclusion criteria. The reasons for exclusion is mentioned in the flowchart (Figure 1). The selected 16 articles were extensively reviewed for their content and their methodology for both qualitative and quantitative analyses (Figure 1).

### 3.2. Characteristics of the Studies

The included articles were published between 2010 and 2020 (Table 2). In all included studies [14,15,16,17,18,19,20,21,22,23,24,25,26,27,28,29], the number of patients ranged from 22 to 86, with a follow-up time of between 6 months and 10 years. The countries, where the studies were carried out were: Turkey [14,16,18,20,27], Brazil [22,23,25], Germany [21,26], Sweden [17,19], Denmark [19], and Saudi Arabia [24]. Ten studies [15,16,17,19,20,21,23,25,27,29] reported that the mean age of the patients was between 7.41 and 55.30 years. Three studies [14,27,28] reported that the patients were children or under 18 years of age. Eight studies [15,16,17,18,19,20,22,25] reported that the total number of patients in relation to their gender (men and women) was 186 and 206, respectively (Table 2).

The total number of treated patients and restored teeth was 764 and 1915, respectively. In 5 studies [17,19,20,21,26], class I and class II restorations were performed, 3 studies [24,27,28] performed class I restorations, 6 studies [14,15,16,18,23,29] performed class II restorations, and 2 studies [22,25] performed non-carious cervical lesions (NCCL) restorations. Among the types of teeth restored, it was observed that the restorations were made in the permanent incisors, canines, premolars, and molars. In two studies [14,27] restorations were performed in primary molars. Regarding the evaluation criteria used for the clinical evaluation of the restorations, all of the studies [14,15,16,17,18,19,20,21,22,23,24,25,26,27,28,29] used the modified parameters of the United States Public Health Service (USPHS) criteria (Table 2).

Six studies [14,18,21,23,26,27] reported that the etching and rinsing method was used and 12 studies [16,17,19,20,21,22,25,26,28,29] used the self-engraving method. The adhesives used in the studies were: Single Bond Universal Adhesive [15,27,29], Clearfil SE Bond [14,22,29], Xeno III [20,21,26], Xeno V [17,19], AdheSE Bond [16,29], OptiBond All-in-One [28,29], Adper Single Bond 2 [18,23], Syntac classic [21,26], XP Bond [23], Tetric N Bond Total-etch [24], Scotchbond Universal Adhesive [25], Excite F [18], Futurabond NR [20], and Peak Universal [23]. The composite resins used in the studies were inserted in the cavities with the following placement techniques: (1) the incremental method, involving Filtek Z550 [14], Charisma Smart Composite [15], Filtek Z350 XT [22], Amelogen Plus [23], Tetric EvoCeram [16,24], Filtek Supreme Ultra Universal [25], Tetric Ceram [21,26], Filtek Z250 [27], Herculite Ultra [28], Clearfil Photo Posterior [29], Ceram X mono [17,19], Filtek Ultimate [18], Grandio [20], and QuiXfil [20]; (2) the bulk method, involving X-tra Fill Bulk [14], Filtek Bulk Fill Posterior Restorative [15,22], Tetric EvoCeram bulk-fill [16,18,24], Filtek Bulk Fill Flowable [25], QuiXfil [21,26], Filtek Bulk-Fill Restorative [27], and Tetric EvoCeram Bulk-Fill [29]; (3) the bulk with sonic activation method, involving SonicFill [14,28,29]; and (4) the two-step bulk method, involving Filtek Bulk Fill Flow + Filtek Z350XT [23], SureFil SDR + TPH3 [23], Filtek Bulk-Fill Flowable + Filtek P60 [29], SDR Flowable + Ceram X Mono [17,19] (Table 2).

Only a few studies [18,21,23,24,28] mentioned using a rubber dam for moisture control during the clinical restorative procedure. Other included studies used cotton rolls and suction for isolation.

The qualitative data synthesis was carried and determined that all studies [14,15,16,17,18,19,20,21,22,23,24,25,26,27,28,29] reported an absence of fractures, absence of discoloration, or marginal staining and adequate marginal adaptation. Of the studies, 15 [14,15,16,17,18,19,20,21,22,24,25,26,27,28,29] reported an absence of post-operative sensitivity; 15 [14,15,16,17,19,20,21,22,23,24,25,26,27,28,29] reported an absence of secondary caries; 14 [14,15,16,17,18,19,20,21,23,24,26,27,28,29] reported adequate color stability and translucency; 13 [15,17,18,19,20,21,22,23,24,25,26,27,28] reported proper surface texture; 13 [14,15,16,17,19,21,22,23,25,26,27,28,29] reported proper anatomical form; 3 [21,23,26] reported adequate tooth integrity without wear and adequate restoration integrity; 2 [21,26] reported proper occlusion; 1 [24] reported the absence of inflammation; and 1 [15] reported an adequate point of contact. The quantitative data on the various clinical parameters extracted from all included studies are provided in Table 3.

### 3.3. Analysis of Risk of Bias of the Studies

**Figure 2:**
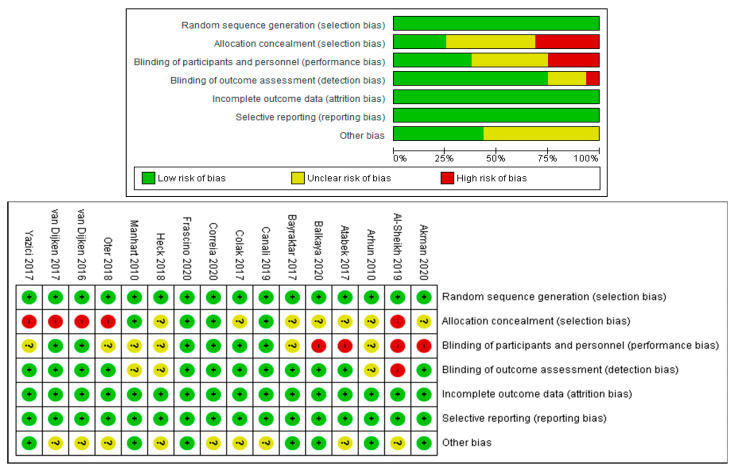
Risk of bias.

Only one study [23] showed a low risk of bias, 1 study [24] showed a high risk of bias, and 14 studies [14,15,16,17,18,19,20,21,22,25,26,27,28,29] showed an unclear (moderate) risk of bias (Figure 2).

### 3.4. Synthesis of Results (Meta-Analysis)

Analysis of the clinical performance of conventional resins and bulk resins in restorations was based on the following 11 parameters: absence of fractures, absence of discoloration or marginal staining, adequate marginal adaptation, absence of post-operative sensitivity, absence of secondary caries, adequate color stability and translucency, proper surface texture, proper anatomical form, adequate tooth integrity (no wear), adequate restoration integrity, and proper occlusion (Appendix A). The other two clinical parameters, absence of inflammation and adequate point of contact, were not analyzed, since each of them was reported by only one included study (Al-Sheikh [24] and Balkaya et al. [15], respectively).

The clinical parameters (modified USPHS criteria) evaluating the clinical effectiveness of conventional resins and bulk resins in restorations were determined in all studies [14,15,16,17,18,19,20,21,22,23,24,25,26,27,28,29], revealing that there were no significant differences between the two types of resins, regardless of the type of restoration, type of tooth restored, or technique used.

The subgroup analysis was performed based on the cavity form (class I/II and non-carious cervical lesions), type of dentition (primary or permanent), and tooth restoration technique (incremental or bulk or two-step bulk). The analyses showed that in the aspect of absence of fractures, absence of discoloration or marginal staining, adequate marginal adaptation, absence of secondary caries, adequate color stability and translucency, proper surface texture, proper anatomical form of the restoration, adequate integrity of the tooth without the presence of wear, adequate restoration integrity, and proper occlusion, there were no significant differences between conventional resins and bulk resins. The data were found to be homogeneous and around the line of no effect (Appendix A).

**Figure A17:**
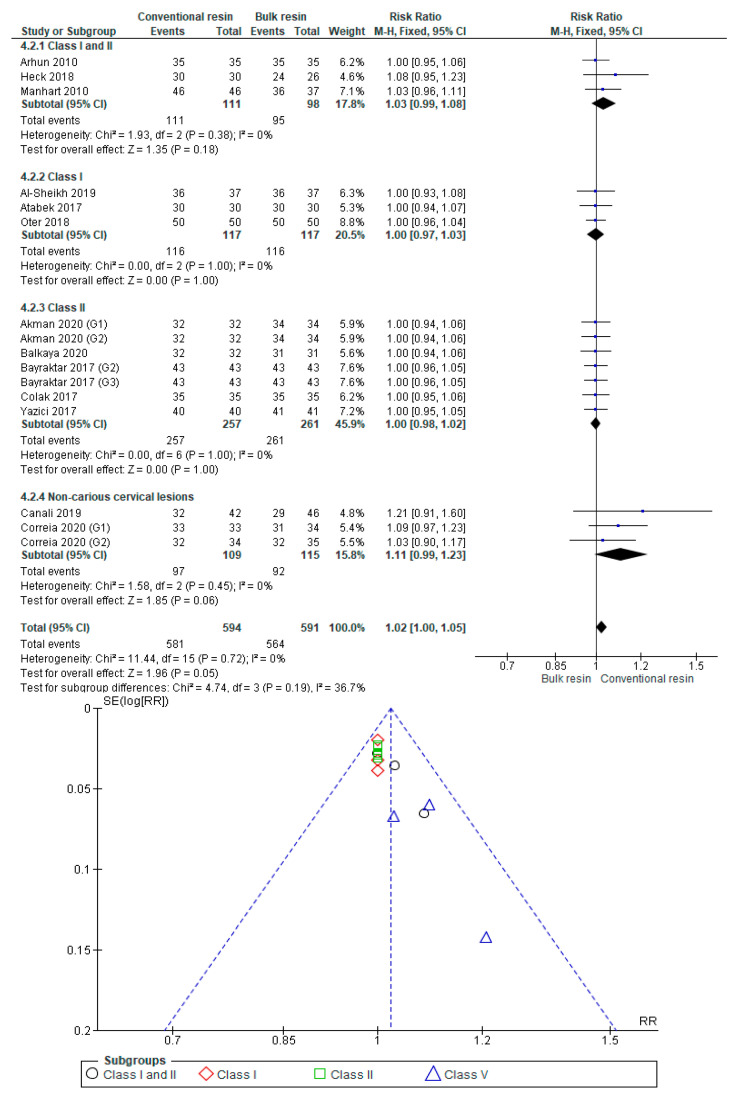
Absence of Post-Operative Sensitivity: Conventional Resin versus Bulk Resin According to Type of Restoration.

**Figure A18:**
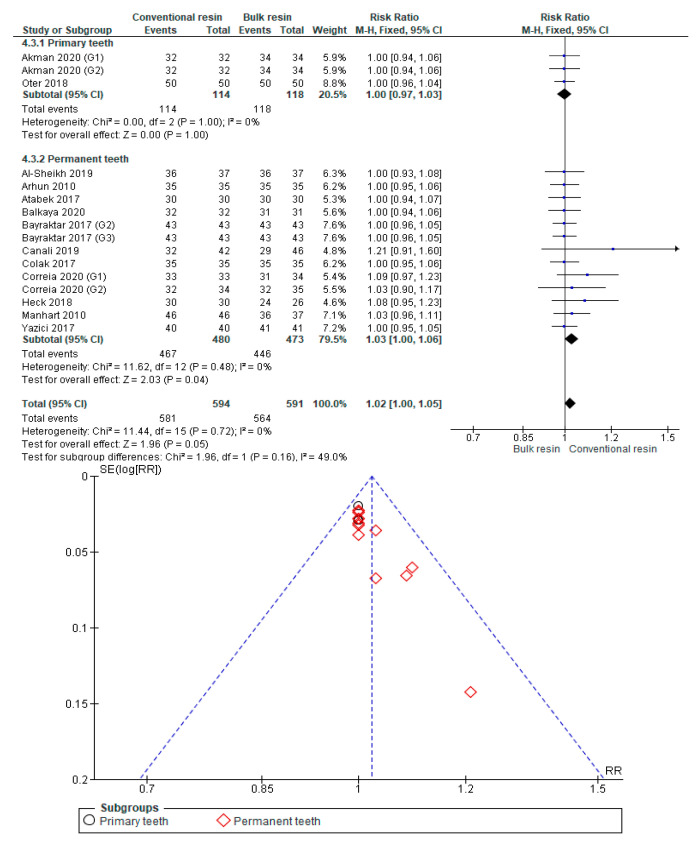
Absence of Post-Operative Sensitivity: Conventional Resin versus Bulk Resin According to Type of Teething.

**Figure A19:**
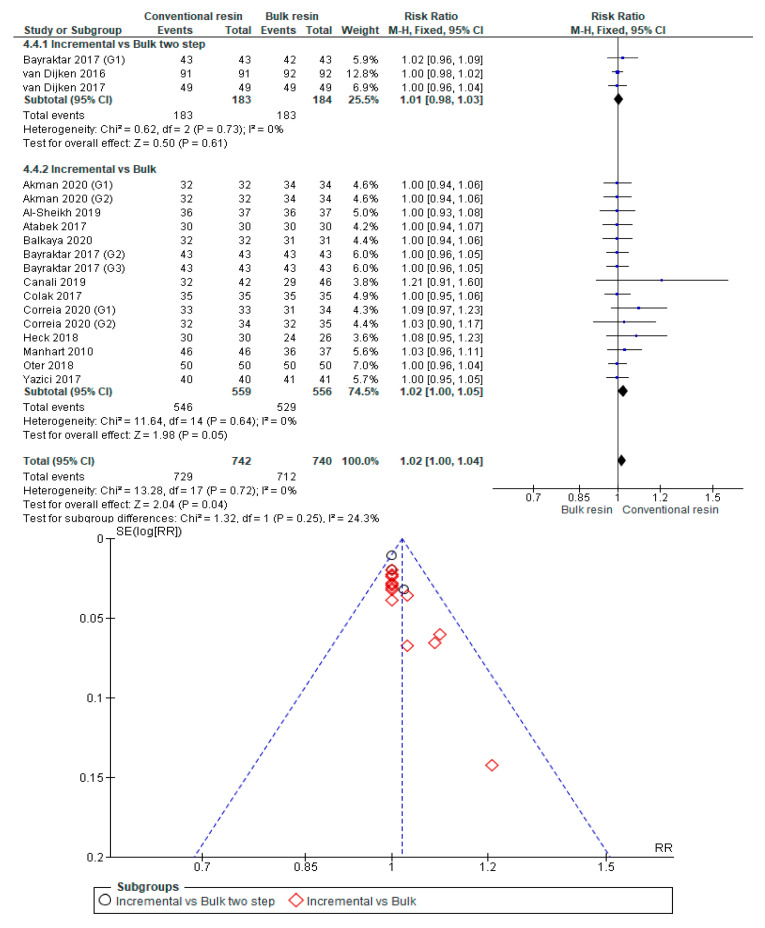
Absence of Post-Operative Sensitivity: Conventional Resin versus Bulk Resin According to the Technique Used.

As for the absence of post-operative sensitivity, the analyses revealed that there were no significant differences when comparing a conventional resin with a bulk resin covered with a conventional resin (two-step bulk technique). However, regarding the clinical feature, there were significant differences between conventional resins and bulk resins in the type of restoration, type of tooth restored, and technique used (Appendix A, Figure A17, Figure A18 and Figure A19). The results were quite interesting, showing reduced or no post-operative sensitivity for NCCLs restored with composite resins rather than bulk ones (RR 1.11 95%CI [0.99, 1.23], *p* = 0.060, with an overall significant effect (RR 1.02 95%CI [1.00, 1.05], *p* = 0.05). A favorable effect of absence in post-operative sensitivity was also seen for cavities treated in permanent dentition (RR 1.03 95%CI [1.00, 1.06], *p* = 0.04) and with incremental technique for composite resins (RR 1.02 95%CI [1.00, 1.05], *p* = 0.05).

## 4. Discussion

In the present investigation, the null hypothesis was not rejected. The clinical effectiveness of bulk-fill resin is similar to conventional resin, regardless of the type of restoration (class I, II, or non-carious cervical lesions), the type of tooth restored (primary or permanent teeth), or the restoration technique used (incremental, bulk, or bulk two step).

The use of restorations based on light-curing composite resins has become widespread due to their adequate mechanical behavior and attractive aesthetic characteristics [30]. These restorations traditionally use a complex restoration technique, which is performed using a so-called “incremental technique” [31,32]. It is used for 2 reasons: (a) the depth of cure of these conventional resin materials is limited, preventing total polymerization in increments that are greater than 2 mm; and (b) an attempt is made to control the effects of material shrinkage when the polymerization reaction occurs [10,31,33,34].

Therefore, for deep or extensive preparations, several layers of the conventional resin material must be applied, which is technically challenging, consumes a lot clinical time, and also involves certain risks, such as the entrapment of air bubbles or contamination between layers. In response to these difficulties, a new generation of composite resins has appeared, called “bulk-fill” resins [10]. The increased translucency of these resins [10,35] due to their incorporation of more photoinitiator reagents [36] allows for deeper photopolymerization and permits insertion of the material into thick 4–5 mm increments, with uniform polymerization and degree of conversion. Furthermore, these resins can be clinically placed via 3 restoration techniques: the two-step bulk technique (using flowable bulk-fill covered with conventional resin material), the bulk technique with sonic activation (using flowable bulk-fill with sonic activation), and the bulk technique (using paste-like or regular bulk-fill) [10]. All of these factors are essential to obtain more satisfactory mechanical properties, and consequently to increase the longevity of the restorations [37,38]. Additionally, bulk-fill resins contain polymerization modulators that achieve low shrinkage and less stress on the bonded interface [35,36,39]. The insertion of thicker increments also contributes to reducing the incorporation of air voids, forming a more homogeneous restorative unit [35,36].

It is known that the longevity of the restorations is related to the clinical situation in the patient’s oral cavity [21,26]. For this reason, there is a greater number of restoration failures in patients with parafunctional habits or temporomandibular disorders. such as bruxism [40]. Van Dijken et al. [17,19] reported a considerable number of failures caused by material or tooth fractures, most of which were in patients with bruxism.

The presence of secondary caries may be associated with the presence of marginal defects in a restoration [41] or with patients with a high risk of caries [42]. Van Dijken et al. [17,19] did not exclude patients with this condition and confirmed that failure caused by secondary caries was associated with patients with a high risk of caries; therefore, secondary caries may be related to biological failure rather than the restorative material used [40,42,43].

The depth [44] and the extension [45] of the cavity are factors that can influence post-operative sensitivity. A single study [28] radiographically confirmed that the restorations were performed in cavities 4–5 mm deep, while the other studies evaluating post-operative sensitivity did not describe the depth of the preparations or did so in more shallow cavities.

In the present review, significant differences were found between bulk resins and conventional resins in their post-operative sensitivity. However, most of the included studies reported either no occurrence of post-operative sensitivity in restored teeth [14,15,16,17,18,19,20,27,28] or no differences in post-operative sensitivity between bulk-fill and conventional resins [24,26,29]. One study argued [16] that the lack of post-operative sensitivity resulted from the placement of liners (e.g., calcium hydroxide liners or resin-modified glass ionomers) in deep and very deep cavities. Another study [28] observed post-operative sensitivity after 12 months in only one tooth of all those restored with bulk-fill with sonic activation, attributing this to the lack of calcium hydroxide liner in the deep cavity. Additionally, one study [18] reported post-operative sensitivity in a bulk-fill-restored tooth, which disappeared at 12-month recall.

Several studies [21,22,25] found that bulk-fill resin placement resulted in higher rates of post-operative sensitivity compared to conventional resins. These studies [22,25] preformed restorations of non-carious cervical lesions using bulk-fill resins and reported significant failure due to post-operative sensitivity within one year follow-up. It was argued that the high scores for post-operative sensitivity resulted from the limitations of the USPHS scale, which only allows the evaluation of either the absence or presence of post-operative sensitivity, without considering the intensity of the symptom. Restoration of NCCL is clinically challenging, as cavities located in the hypersensitive cervical region are more susceptible to contamination. Hence, using universal adhesives with proven antibacterial activity [46,47] and low cytotoxic effect [48] would seem appropriate. However, the clinical performance of bulk-fill resins placed in NCCLS was acceptable, yielding longer than 1-year follow-up periods.

The included studies mostly used universal adhesives in self-etching mode when placing bulk-fill resins. These adhesives are gaining popularity among clinicians, allowing for simplified procedures, however their dentin bonding potential can be enhanced by modifying the application method [49,50]. One study [21] using the self-etch adhesive Xeno III attributed the post-operative sensitivity to the adhesive used rather than to the bulk-fill resin material. A recent study showed no statistically significant relationship between post-operative sensitivity and the bonding system used [51]. Moreover, no association was found between the technique used to place the bulk-fill resin (incremental and bulk) and the depth of the cavity in terms of post-operative sensitivity [45]. A Cochrane review found inconsistent evidence regarding the use of linings and restoration failures, particularly regarding post-operative sensitivity [52].

This meta-analysis showed that there were no significant differences between conventional and bulk-fill resin compounds in terms of the type of restoration, the type of tooth restored, and the technique used. The results of this systematic review and meta-analysis are similar to those of Veloso et al. [53] and Boaro et al. [54]. Both studies reported that the clinical performance of conventional and bulk-fill resin compounds in direct posterior tooth restorations was similar, within a follow-up period of 12 to 72 months and up to 10 years, respectively. These clinical findings might be explained by corresponding mechanical properties, i.e., the shrinkage stress (for regular consistency materials), flexural strength, and fracture strength [54]. However, some of the chemical–physical properties (shrinkage, polymerization stress, cup deflection, and microhardness) of bulk-fill resin composites were found to be superior when compared with conventional composites, regardless of the viscosity or application technique [54]. As for the degree of conversion of bulk-fill materials with flowable consistency (used in two-step bulk technique), this was similar to conventional composite resins with thicknesses of up to 2 mm and greater than conventional composites with thicknesses greater than 2 mm [54].

Another study [55] testing wear and microhardness found that bulk-fill resin materials have comparable microhardness to conventional ones and present minimal change in surface roughness upon wear. This in turn could decrease bacterial adhesion to the surface of the restoration. However, more than the surface roughness, the filler particle size might play a role in bacterial adhesion, showing greater adhesion to resin materials with larger filler particles [56]. The hardness and wear resistance of the composite resins seem to be linked, yet it is difficult to assess resin material behaviour upon ageing [57].

Given that there are no reported clinical differences between restorations made of conventional resin materials and bulk-fill resin materials (in two-step or bulk techniques), these results seems promising, as most clinicians prefer to work with easy-to-use, clinically reliable bulk-fill resin materials, the placement of which occupies less chair-time in the dental office.

The current study has some limitations, such as the design of the clinical trial and the follow-up period, which could influence the results of the clinical trial. Randomized clinical trials are a critical method of clinically evaluating new materials and treatments, because these studies are standardized to achieve greater clinical credibility and reliability. However, a detailed description and similar methods should be used to allow a comparison. The clinical trials included in this study used different bulk-fill restorative materials with different etching techniques, which made it more difficult to compare them.

All of the studies included in this review used the modified USPHS criteria, however there were some differences between each one, resulting in a lack of standardization. The USPHS [58] criteria are the most common criteria for clinical evaluation of restorations. However, they have shown limited sensitivity and the categories may not fully reflect the clinical success of the restorations [59]. Clinical trials that have used other criteria tend to detect failure rates more than 4 times higher than those produced by the USPHS. However, this may be due not only to the increased sensitivity of these criteria, but also to a host of other factors, such as the fact that not all new systems are fully validated.

Likewise, randomization and allocation concealment are critical to the design of randomized clinical trials to avoid selection bias. Most of the included studies did not provide a complete description of these steps. Göstemeyer et al. [60] reviewed the design and validity of randomized clinical trials dealing with dental restorations and observed a high risk of bias, mainly in the domains of allocation concealment (93% selection bias) and blinding of participants and staff (99% performance bias) or blinding of outcome assessment (46% detection bias). Blinding of the operator and examiners may, in certain cases, be more difficult or even impossible to do, depending on the materials studied. However, allocation concealment can be implemented in all trials.

The follow-up periods observed in this study ranged from 6 months to 10 years. Differences in the efficacy of therapies can be measured only after several years because failure behavior can vary, and because one type of material may be more susceptible to long-term dental fractures and the other to secondary caries. Therefore, long observation periods are essential to observe all relevant effects and differences. However, maintaining a population of participants for an extended period is extremely difficult and attrition bias is very common [61].

For all of these reasons, the authors recommend that the results of this review should be interpreted with caution. Additional randomized clinical trials with better designs are needed.

## 5. Conclusions

In light of the current evidence, the clinical performance of conventional resins and bulk resins for carious lesion restorations is similar. However, properly designed clinical studies are required to avoid the biases observed in this study in order to reach a better conclusion.

## Figures and Tables

**Table 1:** The inclusion and exclusion criteria for articles.

Inclusion Criteria	Exclusion Criteria
Studies carried out on vital human teeth. Only randomized clinical trials (RCT) Clinical studies evaluating direct restorations in teeth restored with bulk-fill and conventional resin composites. Prospective studies. Studies with a follow-up time greater than or equal to 6 months.	Studies on class III and class IV cavities. Literature not published in peer-reviewed journals. The gray literature, i.e., the information not reported in the scientific journals. All papers in other than the English language, where the full text was not available. Prospective studies without randomization and retrospective studies. Studies evaluating only bulk-filled resin restorations, without direct comparison with conventional resin composites. Same data that was published at different times.

**Table 2:** Characteristics of included studies.

Author(s)	Year	Country	Type of Study	Number of Patients (Men/Women)	Number of Teeth Restored	Average Age (Range)	Follow-up	Groups	Patients Per Group	Teeth Per Group	Restoration Type	Tooth Type	Evaluation Criteria	Engraving Method	Adhesive	Resin	Placement Technique
Akman et al. [14]	2020	Turkey	RCT parallel double-blind	30	160	(6–10)	1 year	Glass ionomer	30	40	Class II	Primary Molar	Modified US Public Health Service	Etching and rinsing - 20% polyacrylic acid (Cavity Conditioner, GC Corp., Japan)	-----------	Equia Fil (GC Corporation, Japan)	Injected
								Bulk Resin	30	40				Self-etch	Clearfil SE Bond (Kuraray, Tokyo Japan)	SonicFill (Kerr Corporation, USA)	Bulk with sonic activation
								Bulk Resin	30	40						X-tra fil (Voco, Germany)	Bulk
								Conventional Resin	30	40						Filtek Z550 (3M ESPE, USA)	Incremental
Balkaya et al. [15]	2020	Turkey	RCT split mouth double-blind	54 (23/31)	109	22 (20–32)	4 years	Conventional Resin	54	37	Class II	Premolar and Molar	Modified US Public Health Service	Self-etch	Single Bond Universal adhesive (3M ESPE, Neuss, Germany)	Charisma Smart Composite (Heraeus Kulzer, Hanau, Germany)	Incremental
								Bulk Resin	54	38						Filtek Bulk Fill Posterior Restorative (3M ESPE, St. Paul, MN, USA)	Bulk
								Glass ionomer	54	34					----------	Equia Forte Fil (GC, Tokyo, Japan)	Injected
Correia et al. [22]	2020	Brazil	RCT parallel double-blind	77 (34/43)	140	(21–80)	1 year	Conventional Resin—1.5 mm OGD	28	35	NCCL	Canine and Premolar	Modified US Public Health Service	Self-etch	Clearfil SE Bond (Kuraray America, Inc, New York, NY, USA)	Filtek Z350 XT (3M ESPE, St Paul, MN, USA)	Incremental
								Bulk Resin—1.5 mm OGD	27	35						Filtek Bulk Fill Posterior (3M ESPE)	Bulk
								Conventional Resin—3 mm OGD	27	35						Filtek Z350 XT (3M ESPE, St Paul, MN, USA)	Incremental
								Bulk Resin—3 mm OGD	29	35						Filtek Bulk Fill Posterior (3M ESPE)	Bulk
Frascino et al. [23]	2020	Brazil	RCT split mouth	53	159	48.3 ± 10	1 year	Conventional Resin	53	53	Class II	Premolar and Molar	Modified US Public Health Service	Etching and rinsing—35% phosphoric acid gel (Ultra-Etch, Ultradent)	Peak Universal (Ultradent, South Jordan, UT, USA)	Amelogen Plus (Ultradent, South Jordan, UT, USA)	Incremental
								Bulk Resin + Conventional Resin	53	53					Adper Single Bond 2 (3M ESPE, St Paul, MN, USA)	Filtek Bulk Fill Flow (3M ESPE, St Paul, MN, USA) + Filtek Z350XT (3M ESPE, St Paul, MN, USA)	Two-step Bulk (4 mm + 2 mm)
								Bulk Resin + Conventional Resin	53	53					XP Bond (Dentsply, Milford, DE, USA)	SureFil SDR (Dentsply, Milford, DE, USA) + TPH3 (Dentsply, Milford, DE, USA)	Two-step Bulk (4 mm + 2 mm)
Al-Sheikh [24]	2019	Saudi Arabia	RCT split mouth	40	80	(20–40)	6 months	Conventional Resin	40	40	Class I	Molar	Modified US Public Health Service	Etching and rinsing—Tetric NEtch (Ivoclar Vivadent, Schaan, Liechtenstein)	Tetric NBond Total-Etch (Ivoclar Vivadent, Schaan, Liechtenstein)	Tetric EvoCeram (Ivoclar Vivadent, Schaan, Liechtenstein)	Incremental
								Bulk Resin	40	40						Tetric EvoCeram bulk-fill (Ivoclar Vivadent, Schaan, Liechtenstein)	Bulk
Canali et al. [25]	2019	Brazil	RCT split mouth double-blind	22 (5/17)	89	41.1 ± 12.7 (21–69)	1 year	Conventional resin	22	43	NCCL	Incisor, canine, premolar and molar	Modified US Public Health Service	Self-etch	Scotchbond Universal Adhesive (3M ESPE, St. Paul, MN)	Filtek Supreme Ultra Universal (3M ESPE, St. Paul, MN)	Incremental
								Bulk resin	22	46						Filtek Bulk Fill Flowable (3M ESPE, St. Paul, MN)	Bulk
Heck et al. [26]	2018	Germany	RCT split mouth	46	96	(>18)	10 years	Conventional resin	46	50	Class I and II	Molar	Modified US Public Health Service	Etching and rising—37% phosphoric acid	Syntac classic (Ivoclar Vivadent, Schaan, Liechtenstein)	Tetric Ceram (Ivoclar Vivadent, Schaan, Liechtenstein)	Incremental
								Bulk resin	46	46				Self-etch	Xeno III (Dentsply De Trey, Konstanz, Germany)	QuiXfil (Dentsply De Trey, Konstanz, Germany)	Bulk
Oter et al. [27]	2018	Turkey	RCT split mouth single-blind	80	160	7.41 ± 1.8	1 year	Conventional resin	80	80	Class I	PrimaryMolar	Modified US Public Health Service	Etching and rising	Single Bond Universal Adhesive (3M, Neuss, Germany)	Filtek Z250 (3M ESPE, St Paul, USA)	Incremental
								Bulk resin	80	80						Filtek Bulk-Fill Restorative (3M ESPE, St Paul, USA).	Bulk
Atabek et al. [28]	2017	Turkey	RCT split mouth	30	60	(7–16)	2 years	Conventional resin	30	30	Class I	Molar	Modified US Public Health Service	Self-etch	OptiBond All-In-One (Kawo Sonic Fill System; Kerr, Orange, USA)	Herculite Ultra (Kerr, Orange, USA)	Incremental
								Bulk resin	30	30						SonicFill (Kawo Sonic Fill System; Kerr, Orange, USA)	Bulk with sonic activation
Bayraktar et al. [29]	2017	Turkey	RCT split mouth	50	200	25.8 ± 7.49 (18–45)	1 year	Conventional resin	50	50	Class II	Premolar and Molar	Modified US Public Health Service	Self-etch	Clearfil SE Bond (Kuraray, Okayama, Japan)	Clearfil Photo Posterior (Kuraray, Okayama, Japan)	Incremental
								Bulk Resin + Conventional Resin	50	50					Single Bond Universal (3M ESPE, St Paul, USA)	Filtek Bulk-Fill Flowable (3M ESPE, St Paul, USA) + Filtek P60 (3M ESPE, St Paul, USA)	Two-step Bulk (2–4 mm + 2 mm)
								Bulk resin	50	50					Adhe SE Bond (Ivoclar Vivadent, Schaan, Liechtenstein)	Tetric EvoCeram Bulk-Fill (Ivoclar Vivadent, Schaan, Liechtenstein)	Bulk
								Bulk resin	50	50					OptiBond All-In-One (Kawo Sonic Fill System; Kerr, Orange, USA)	SonicFill (Kawo Sonic Fill System; Kerr, Orange, USA)	Bulk with sonic activation
Colak et al. [16]	2017	Turkey	RCT split mouth double–blind	34 (24/10)	74	33.74 ± 6.8 (23–56)	1 year	Conventional resin	34	37	Class II	Premolar and Molar	Modified US Public Health Service	Self-etch	AdheSE Bond (Ivoclar Vivadent, Schaan, Liechtenstein)	Tetric EvoCeram (Ivoclar Vivadent, Schaan, Liechtenstein)	Incremental
								Bulk resin	34	37						Tetric EvoCeram bulk-fill (Ivoclar Vivadent, Schaan, Liechtenstein)	Bulk
van Dijken et al. [17]	2017	Sweden	RCT split mouth double blind	38 (22/16)	106	55.3 (32–87)	6 years	Conventional resin	38	53	Class I and II	Premolar and Molar	Modified US Public Health Service	Self-etch	XenoV (Dentsply Sirona, Konstanz, Germany)	Ceram X mono (DentsplySirona, Konstanz, Germany)	Incremental
								Bulk Resin + Conventional Resin	38	53						SDR flowable (DentsplySirona, Konstanz, Germany) + Ceram X mono (DentsplySirona, Konstanz, Germany)	Two-step Bulk (4 mm + 2 mm)
Yazici et al. [18]	2017	Turkey	RCT split mouth double-blind	50 (24/26)	104	(24–55)	3 years	Conventional resin	50	52	Class II	Premolar and Molar	Modified US Public Health Service	Etching and rising	Adper Single Bond 2 (3M ESPE, St Paul, USA)	Filtek Ultimate (3M ESPE, St Paul, USA)	Incremental
								Bulk resin	50	52					Excite F (Ivoclar Vivadent, Schaan, Liechtenstein)	Tetric EvoCeram Bulk Fill (Ivoclar Vivadent, Schaan, Liechtenstein)	Bulk
van Dijken et al. [19]	2016	Sweden and Denmark	RCT split mouth double-blind	86 (44/42)	200	52.4 (20–86)	5 years	Conventional resin	86	100	Class I and II	Premolar and Molar	Modified US Public Health Service	Self-etch	XenoV (Dentsply/DeTrey, Konstanz, Germany)	Ceram X mono (Dentsply/DeTrey, Konstanz, Germany)	Incremental
								Bulk Resin + Conventional Resin	86	100						SDR flowable (Dentsply/DeTrey, Konstanz, Germany) + Ceram X mono (Dentsply/DeTrey, Konstanz, Germany)	Two-step Bulk (4 mm + 2 mm)
Arhun et al. [20]	2010	Turkey	RCT split mouth	31(10/21)	82	36 (16–60)	2 years	Conventional resin	31	41	Class I and II	Premolar and Molar	Modified US Public Health Service	Self-etch	Futurabond NR (Voco GmbH, Cuxhaven, Germany)	Grandio (Voco GmbH, Cuxhaven, Germany)	Incremental
								Bulk resin	31	41					Xeno III (Dentsply Caulk, Milford, DE, USA)	QuiXfil (Dentsply Caulk, Milford, DE, USA)	Incremental
Manhart et al. [21]	2010	Germany	RCT split mouth	43	96	44.3 (19–67)	4 years	Conventional resin	43	50	Class I and II	Molar	Modified US Public Health Service	Etching and rising - 37% phosphoric acid	Syntac classic (Ivoclar Vivadent, Schaan, Liechtenstein)	Tetric Ceram (Ivoclar Vivadent, Schaan, Liechtenstein)	Incremental
								Bulk resin	43	46				Self-etch	Xeno III (Dentsply De Trey, Konstanz, Germany)	QuiXfil (Dentsply De Trey, Konstanz, Germany)	Bulk

Legend: OGD = occlusogingival distance; NCCL = non-carious cervical lesions; NR = nor reported; RCT = randomized clinical trial.

**Table 3:** Data extracted from included studies.

Author(s)	Clinical Parameters												
	Absence of Fractures	Absence of Discoloration or Marginal Staining	Adequate Marginal Adaptation	Absence of Post-Operative Sensitivity	Absence of Secondary Caries	Adequate Color, Stability, and Translucency	Proper Surface Texture	Proper Anatomical Form	Adequate Tooth Integrity/No Wear	Adequate Restoration Integrity	Proper Occlusion	Absence of Inflammation	Adequate Point of Contact
Akman et al. (2020) [14]	28/34	30/34	28/34	34/34	34/34	34/34	NR	34/34	NR	NR	NR	NR	NR
	34/34	32/34	33/34	34/34	34/34	34/34	NR	34/34	NR	NR	NR	NR	NR
	34/34	32/34	33/34	34/34	34/34	34/34	NR	34/34	NR	NR	NR	NR	NR
	32/32	30/32	31/32	32/32	32/32	32/32	NR	32/32	NR	NR	NR	NR	NR
Balkaya et al. (2020) [15]	32/32	31/32	23/32	32/32	32/32	32/32	30/32	32/32	NR	NR	NR	NR	32/32
	31/31	29/31	27/31	31/31	31/31	31/31	31/31	31/31	NR	NR	NR	NR	31/31
	15/21	20/21	10/21	21/21	21/21	5/21	11/21	15/21	NR	NR	NR	NR	14/21
Correia et al. (2020) [22]	33/33	29/33	32/33	33/33	33/33	NR	32/33	33/33	NR	NR	NR	NR	NR
	33/34	29/34	33/34	31/34	33/34	NR	32/34	33/34	NR	NR	NR	NR	NR
	34/34	25/34	34/34	32/34	34/34	NR	32/34	34/34	NR	NR	NR	NR	NR
	34/35	31/35	33/35	32/35	34/35	NR	33/35	34/35	NR	NR	NR	NR	NR
Frascino et al. (2020) [23]	50/53	39/53	38/53	NR	53/53	45/53	51/53	52/53	52/53	45/53	NR	NR	NR
	52/53	39/53	39/53	NR	52/53	49/53	50/53	52/53	52/53	35/53	NR	NR	NR
	51/53	41/53	44/53	NR	52/53	51/53	49/53	53/53	52/53	50/53	NR	NR	NR
Al-Sheikh (2019) [24]	37/38	37/37	37/37	36/37	37/37	37/37	37/37	NR	NR	NR	NR	37/37	NR
	37/39	35/37	37/37	36/37	36/37	37/37	36/37	NR	NR	NR	NR	37/37	NR
Canali et al. (2019) [25]	42/43	42/42	26/42	32/42	42/42	NR	30/42	36/42	NR	NR	NR	NR	NR
	46/46	46/46	28/46	29/46	46/46	NR	43/46	40/46	NR	NR	NR	NR	NR
Heck et al. (2018) [26]	29/30	20/30	22/30	30/30	27/30	30/30	29/30	30/30	27/30	30/30	30/30	NR	NR
	22/26	12/26	14/26	24/26	24/26	25/26	24/26	25/26	21/26	19/26	24/26	NR	NR
Oter et al. (2018) [27]	50/50	43/50	47/50	50/50	50/50	45/50	48/50	45/50	NR	NR	NR	NR	NR
	50/50	37/50	45/50	50/50	50/50	44/50	49/50	45/50	NR	NR	NR	NR	NR
Atabek et al. (2017) [28]	30/30	29/30	29/30	30/30	30/30	28/30	28/30	30/30	NR	NR	NR	NR	NR
	30/30	29/30	30/30	30/30	30/30	29/30	29/30	30/30	NR	NR	NR	NR	NR
Bayraktar et al. (2017) [29]	43/43	42/43	43/43	43/43	42/43	43/43	NR	43/43	NR	NR	NR	NR	NR
	42/43	41/43	40/43	42/43	41/43	42/43	NR	40/43	NR	NR	NR	NR	NR
	43/43	42/43	42/43	43/43	41/43	43/43	NR	42/43	NR	NR	NR	NR	NR
	43/43	43/43	43/43	43/43	43/43	43/43	NR	43/43	NR	NR	NR	NR	NR
Colak et al. (2017) [16]	35/35	31/35	34/35	35/35	35/35	35/35	NR	35/35	NR	NR	NR	NR	NR
	35/35	34/35	35/35	35/35	35/35	34/35	NR	35/35	NR	NR	NR	NR	NR
van Dijken et al. (2017) [17]	46/49	46/49	36/49	49/49	49/49	6/49	46/49	42/49	NR	NR	NR	NR	NR
	46/49	44/49	39/49	49/49	48/49	2/49	45/49	43/49	NR	NR	NR	NR	NR
Yazici et al. (2017) [18]	40/40	32/40	30/40	40/40	NR	38/40	38/40	NR	NR	NR	NR	NR	NR
	41/41	39/41	37/41	41/41	NR	41/41	41/41	NR	NR	NR	NR	NR	NR
van Dijken et al. (2016) [19]	86/91	73/91	79/91	91/91	89/91	36/91	85/91	80/91	NR	NR	NR	NR	NR
	89/92	68/92	69/92	92/92	90/92	35/92	83/92	84/92	NR	NR	NR	NR	NR
Arhun et al. (2010) [20]	35/37	35/35	30/35	35/35	35/35	32/35	26/35	NR	NR	NR	NR	NR	NR
	35/37	33/35	31/35	35/35	35/35	35/35	34/35	NR	NR	NR	NR	NR	NR
Manhart et al. (2010) [21]	45/46	34/46	41/46	46/46	46/46	45/46	44/46	45/46	44/46	45/46	45/46	NR	NR
	34/37	26/37	33/37	36/37	37/37	37/37	35/37	36/37	32/37	34/37	35/37	NR	NR

Legend: NR = not reported.

**Figure A1:**
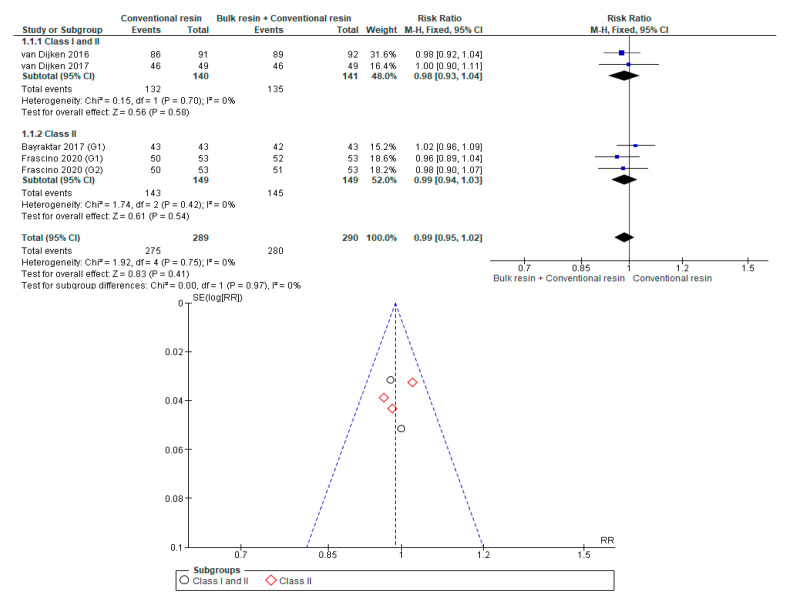
Absence of Fractures: Conventional Resin versus Bulk Resin Covered with Conventional Resin According to Type of Restoration.

**Figure A2:**
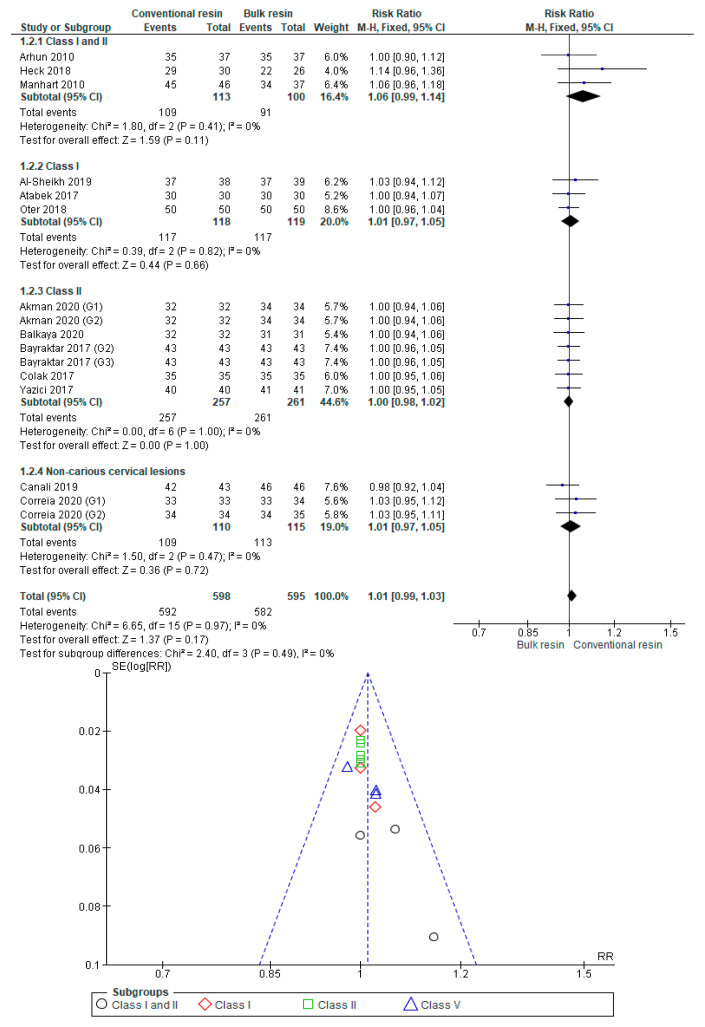
Absence of Fractures: Conventional Resin versus Bulk Resin According to Type of Restoration.

**Figure A3:**
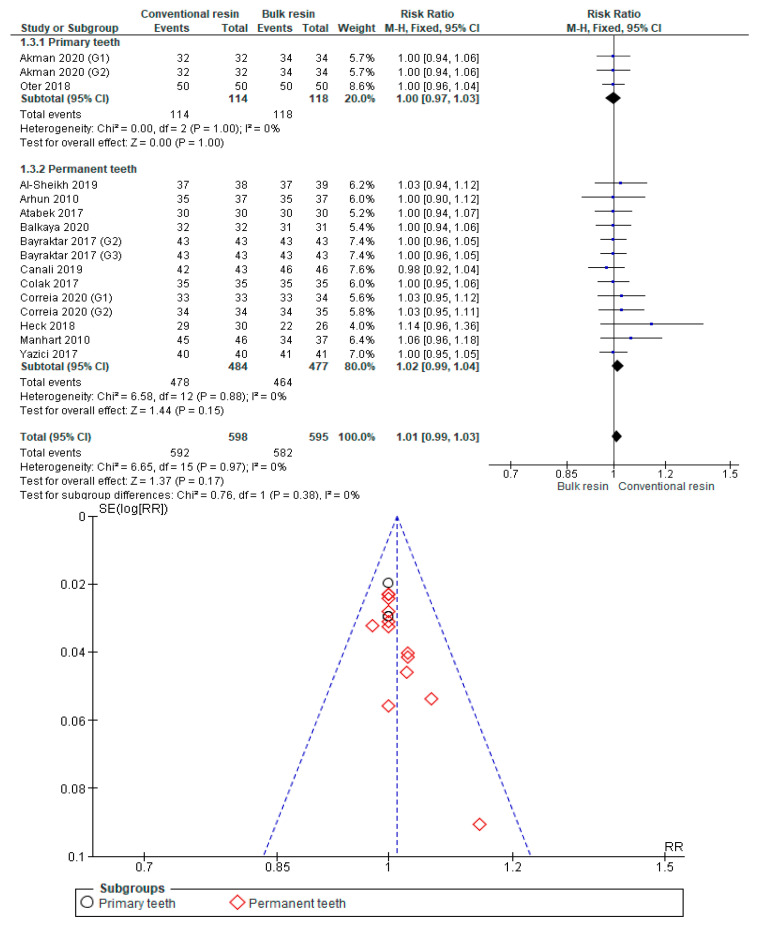
Absence of Fractures: Conventional Resin versus Bulk Resin According to Type of Teething.

**Figure A4:**
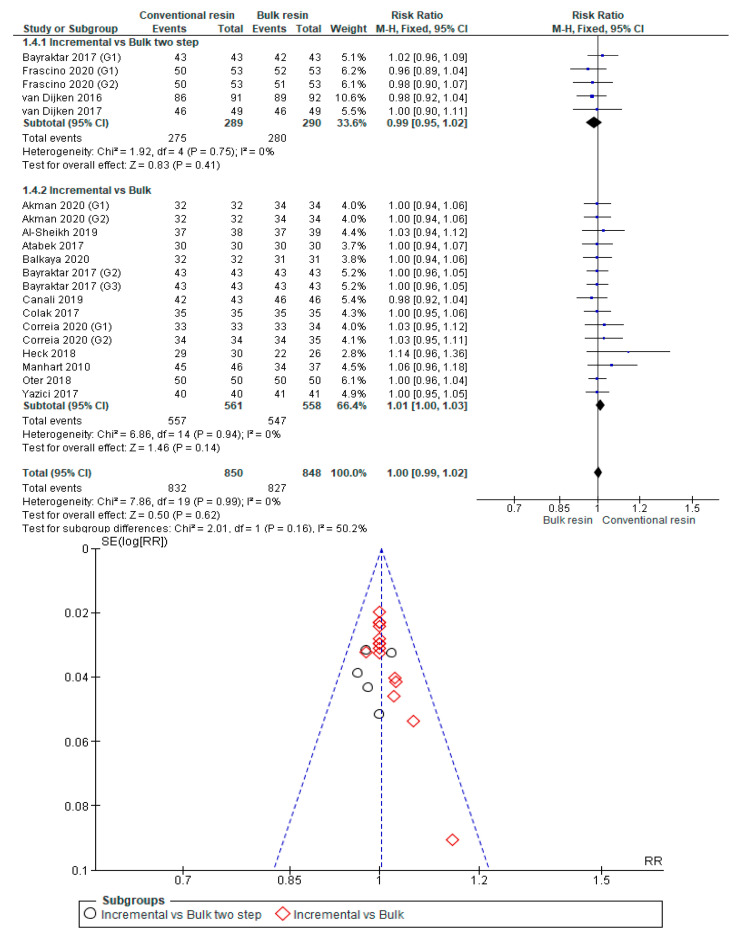
Absence of Fractures: Conventional Resin versus Bulk Resin According to the Technique Used.

**Figure A5:**
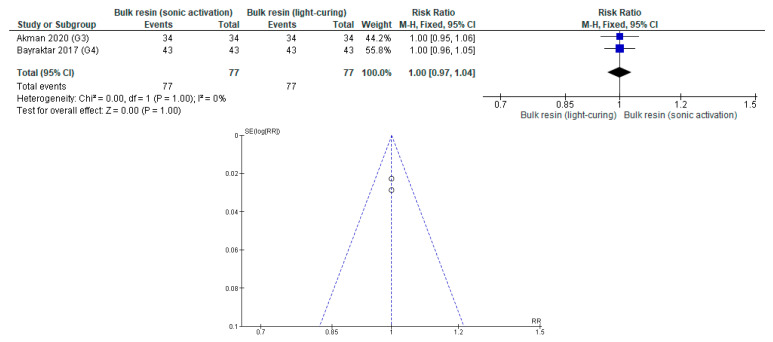
Absence of Fractures: Bulk Resin (with Sonic Activation) versus Bulk Resin (Two-Step Technique).

**Figure A6:**
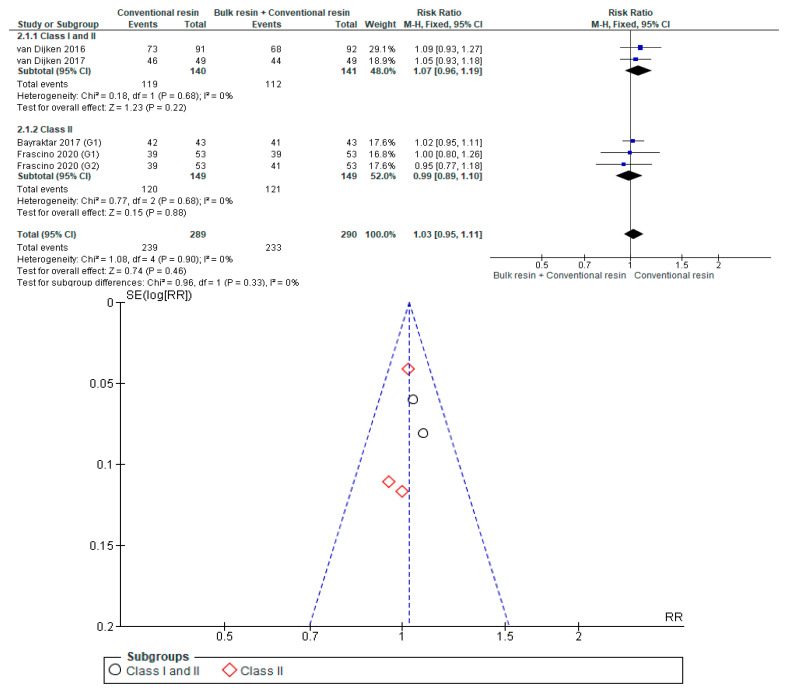
Absence of Discoloration or Marginal Staining: Conventional Resin versus Bulk Resin Covered with Conventional Resin According to Type of Restoration.

**Figure A7:**
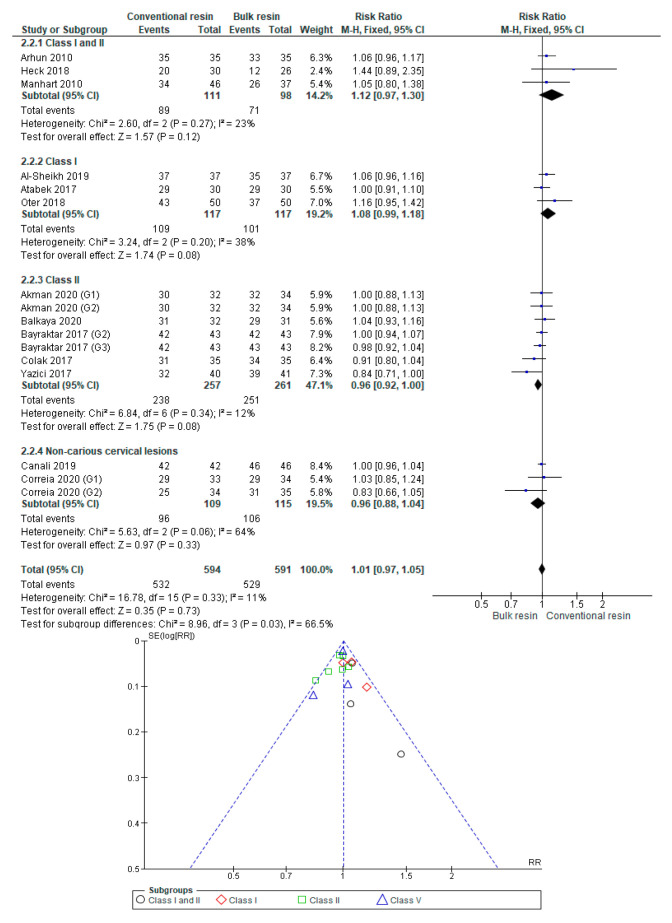
Absence of Discoloration or Marginal Staining: Conventional Resin versus Bulk Resin According to Type of Restoration.

**Figure A8:**
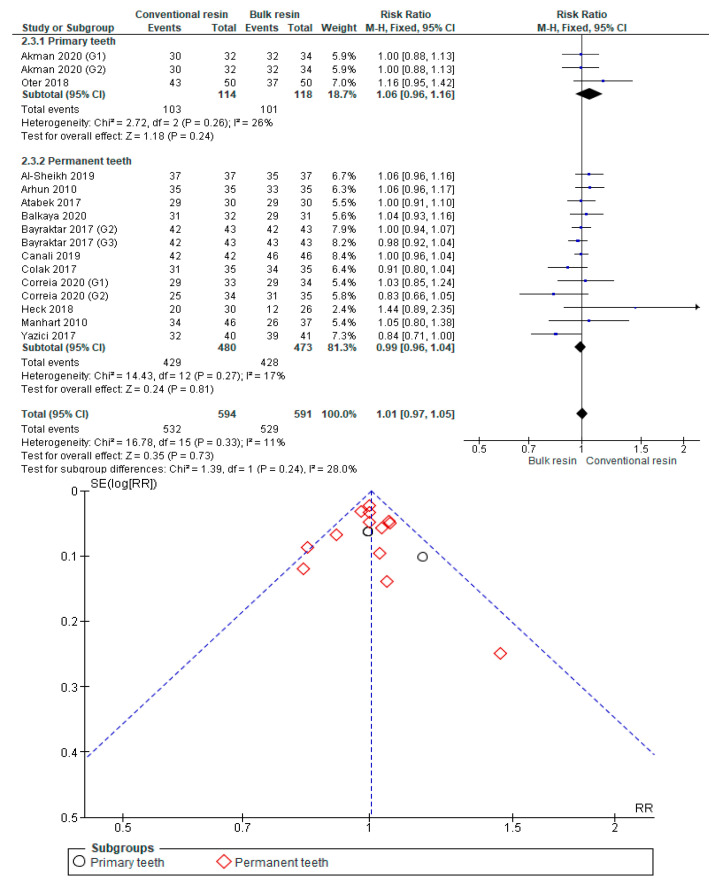
Absence of Discoloration or Marginal Staining: Conventional Resin versus Bulk Resin According to Type of Teething.

**Figure A9:**
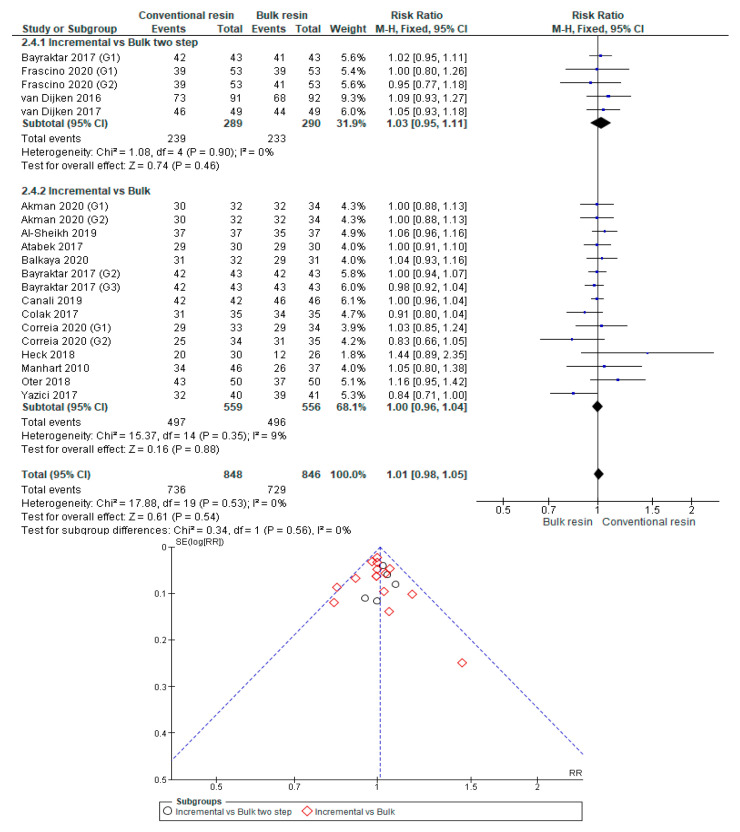
Absence of Discoloration or Marginal Staining: Conventional Resin versus Bulk Resin According to the Technique Used.

**Figure A10:**
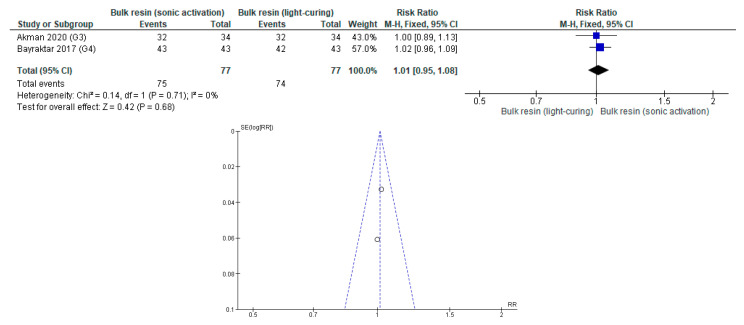
Absence of Discoloration or Marginal Staining: Bulk Resin (with Sonic Activation) versus Bulk Resin (Two-Step Technique).

**Figure A11:**
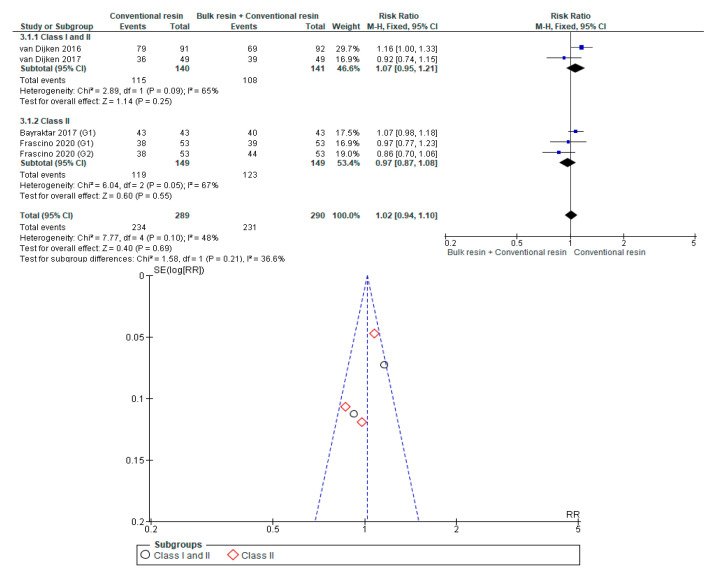
Adequate Marginal Adaptation: Conventional Resin versus Bulk Resin Covered with Conventional Resin According to Type of Restoration.

**Figure A12:**
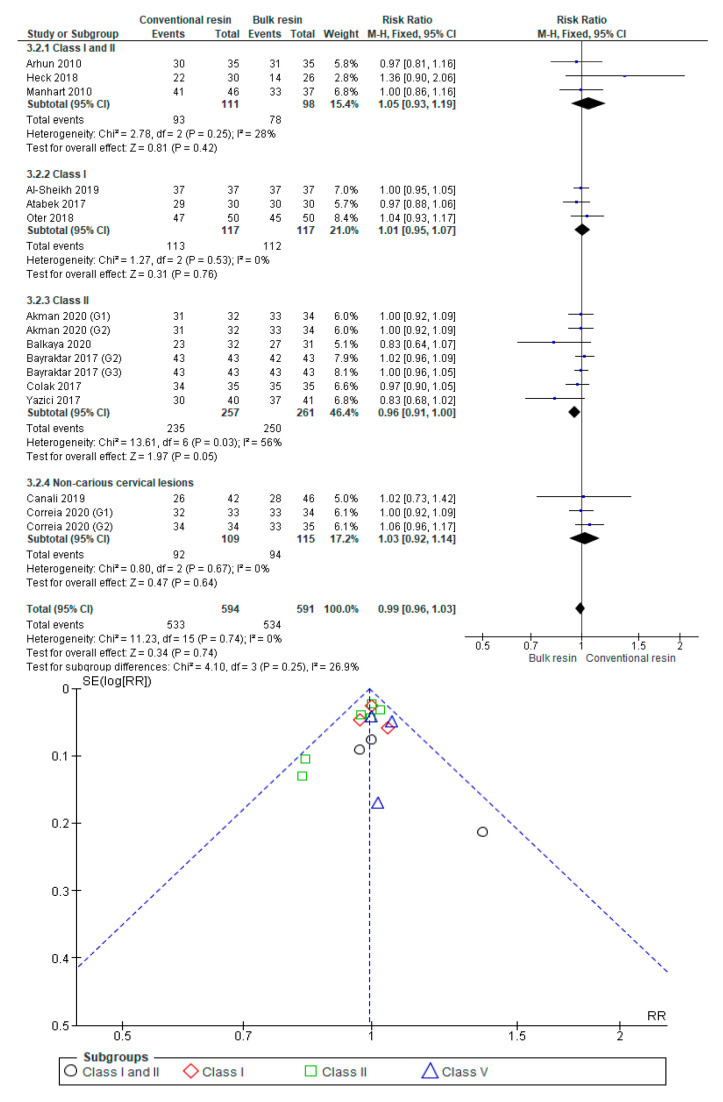
Adequate Marginal Adaptation: Conventional Resin versus Bulk Resin According to Type of Restoration.

**Figure A13:**
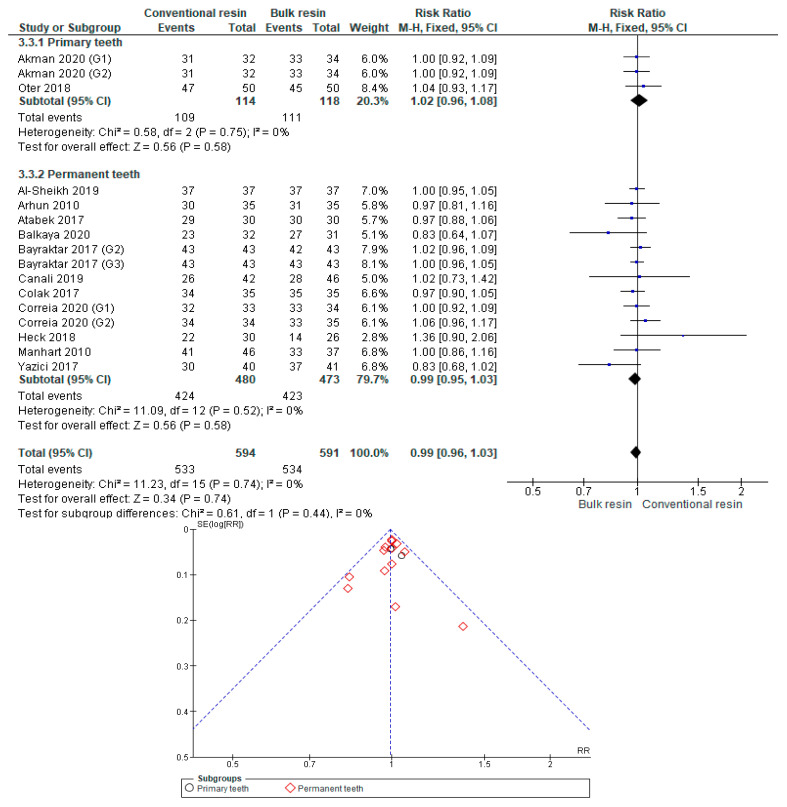
Adequate Marginal Adaptation: Conventional Resin versus Bulk Resin According to Type of Teething.

**Figure A14:**
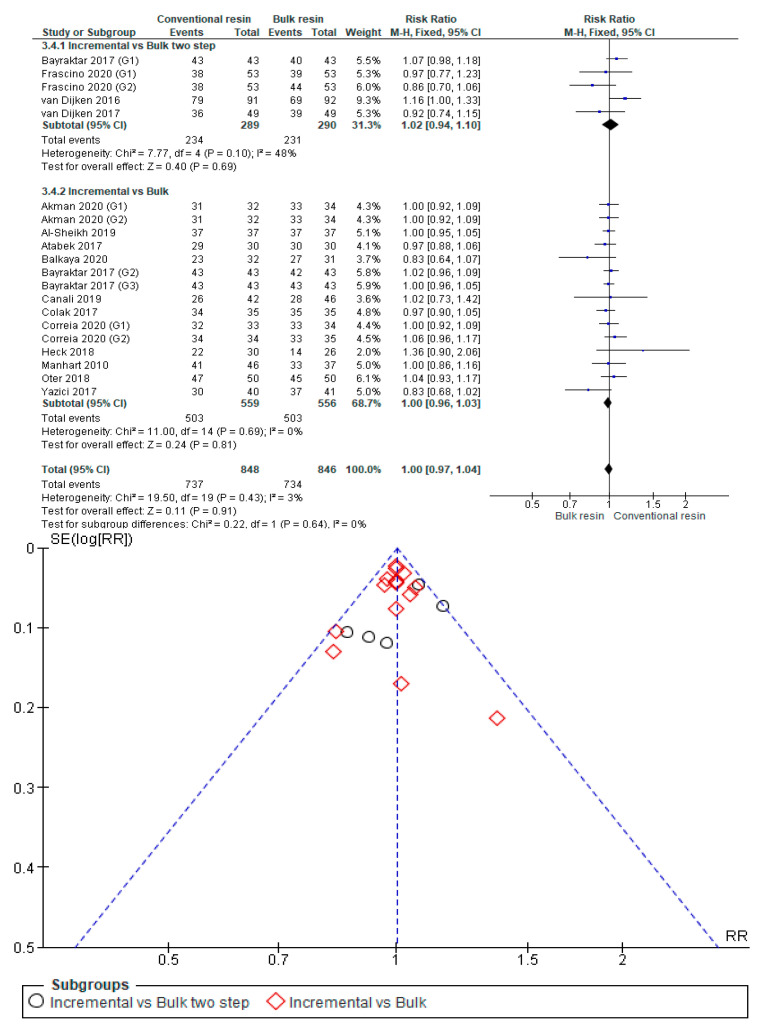
Adequate Marginal Adaptation: Conventional Resin versus Bulk Resin According to the Technique Used.

**Figure A15:**
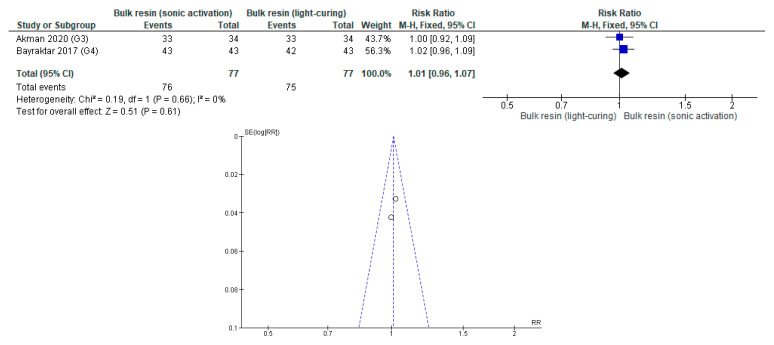
Adequate Marginal Adaptation: Bulk Resin (with Sonic Activation) versus Bulk Resin (Two-Step Technique).

**Figure A16:**
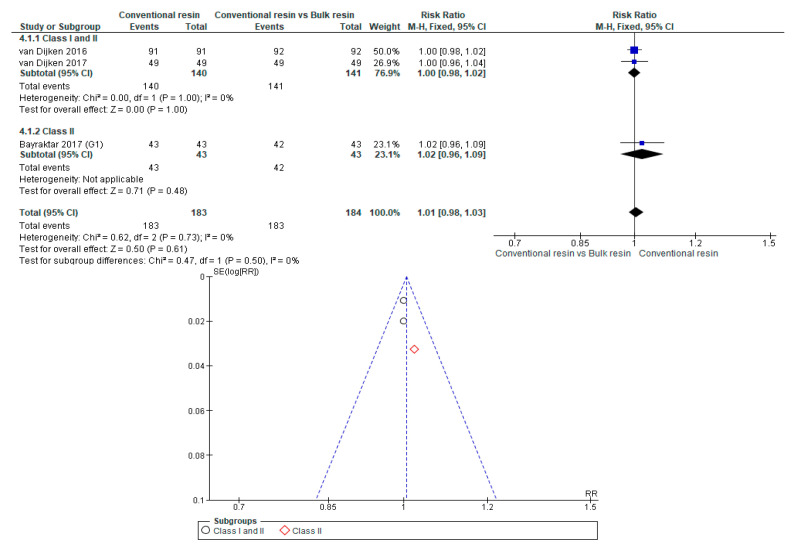
Absence of Post-Operative Sensitivity: Conventional Resin versus Bulk Resin Covered with Conventional Resin According to Type of Restoration.

**Figure A20:**
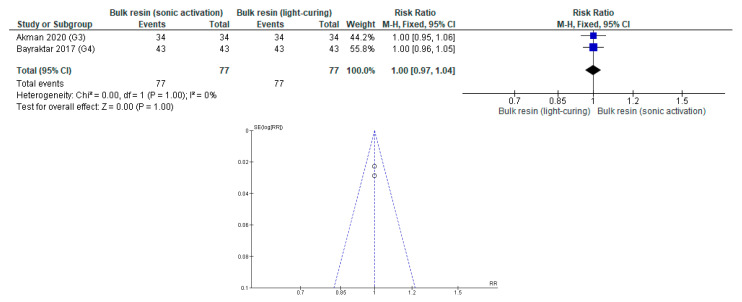
Absence of Post-Operative Sensitivity: Bulk Resin (with Sonic Activation) versus Bulk Resin (Two-Step Technique).

**Figure A21:**
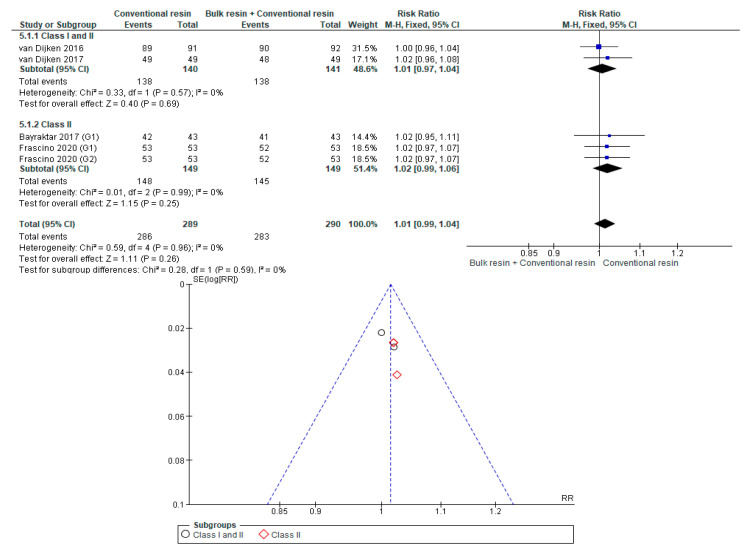
Absence of Secondary Caries: Conventional Resin versus Bulk Resin Covered with Conventional Resin According to Type of Restoration.

**Figure A22:**
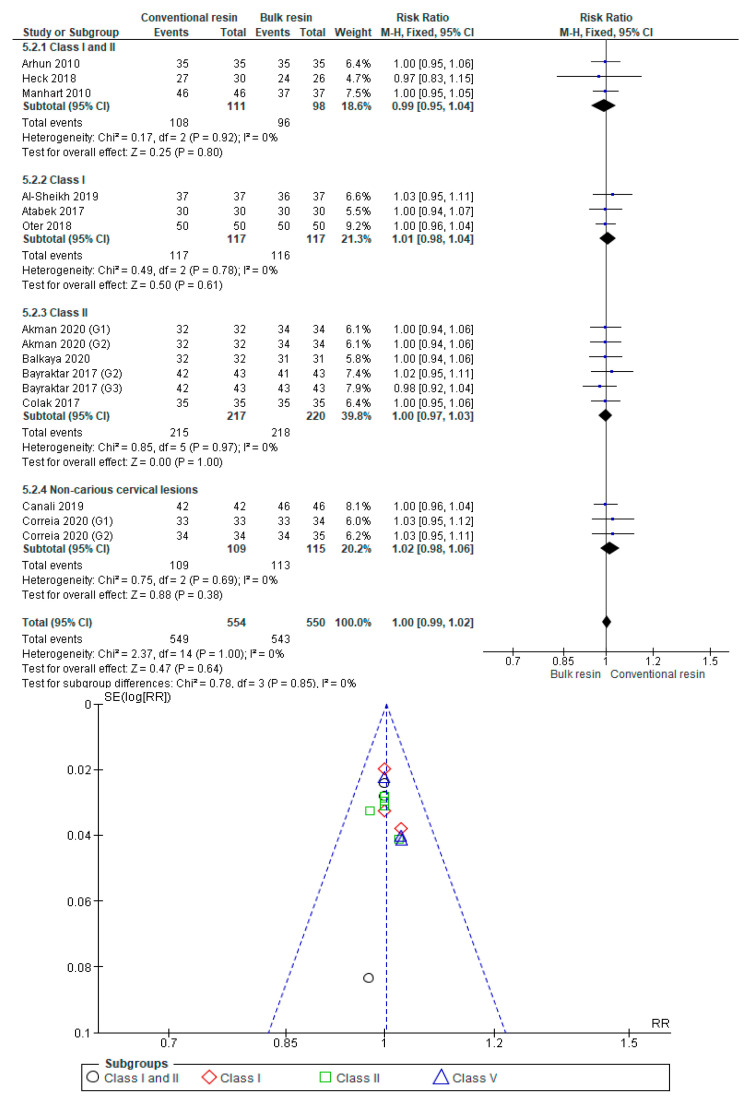
Absence of Secondary Caries: Conventional Resin versus Bulk Resin According to Type of Restoration.

**Figure A23:**
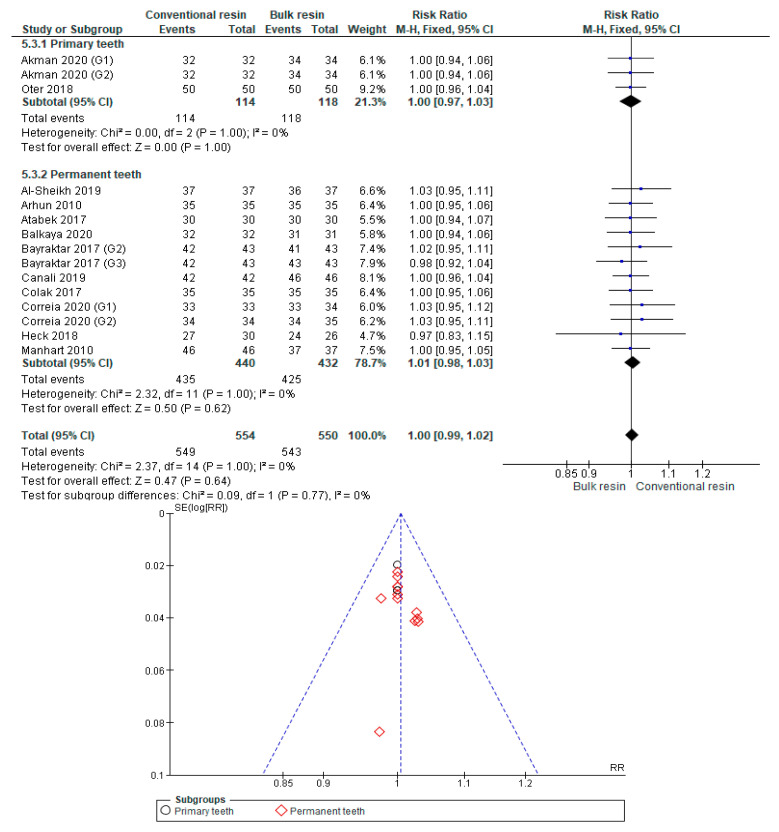
Absence of Secondary Caries: Conventional Resin versus Bulk Resin According to Type of Teething.

**Figure A24:**
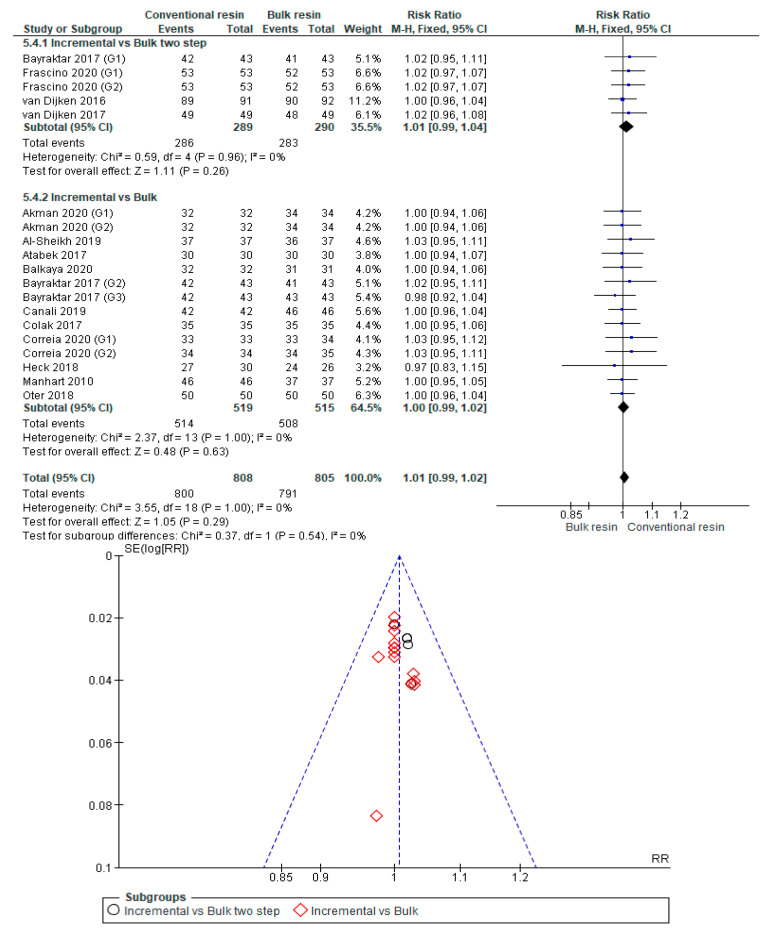
Absence of Secondary Caries: Conventional Resin versus Bulk Resin According to the Technique Used.

**Figure A25:**
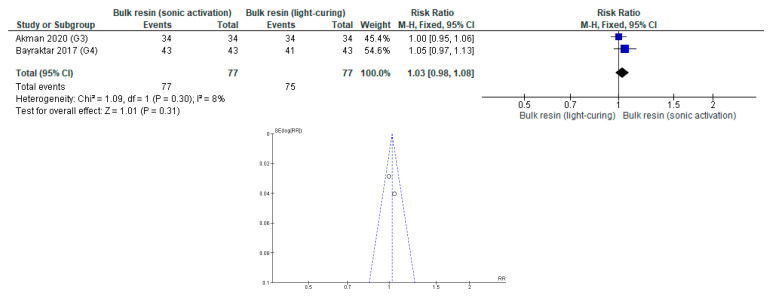
Absence of Secondary Caries: Bulk Resin (with Sonic Activation) versus Bulk Resin (Two-Step Technique).

**Figure A26:**
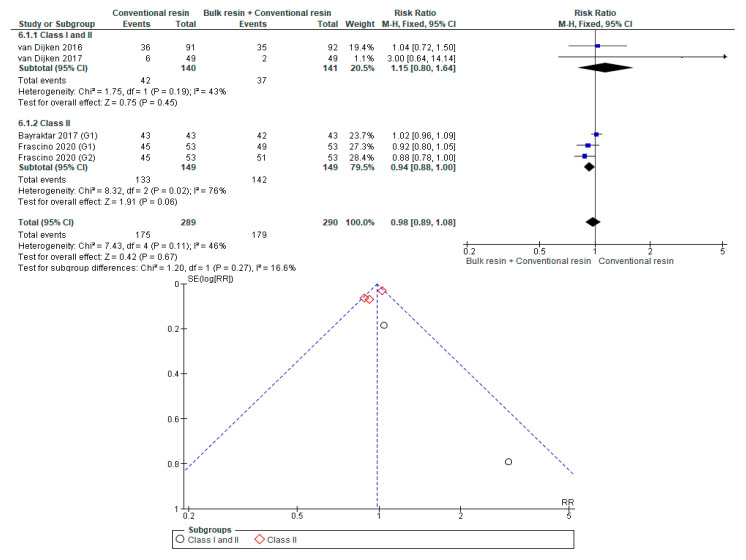
Adequate Color Stability and Translucency: Conventional Resin versus Bulk Resin Covered with Conventional Resin According to Type of Restoration.

**Figure A27:**
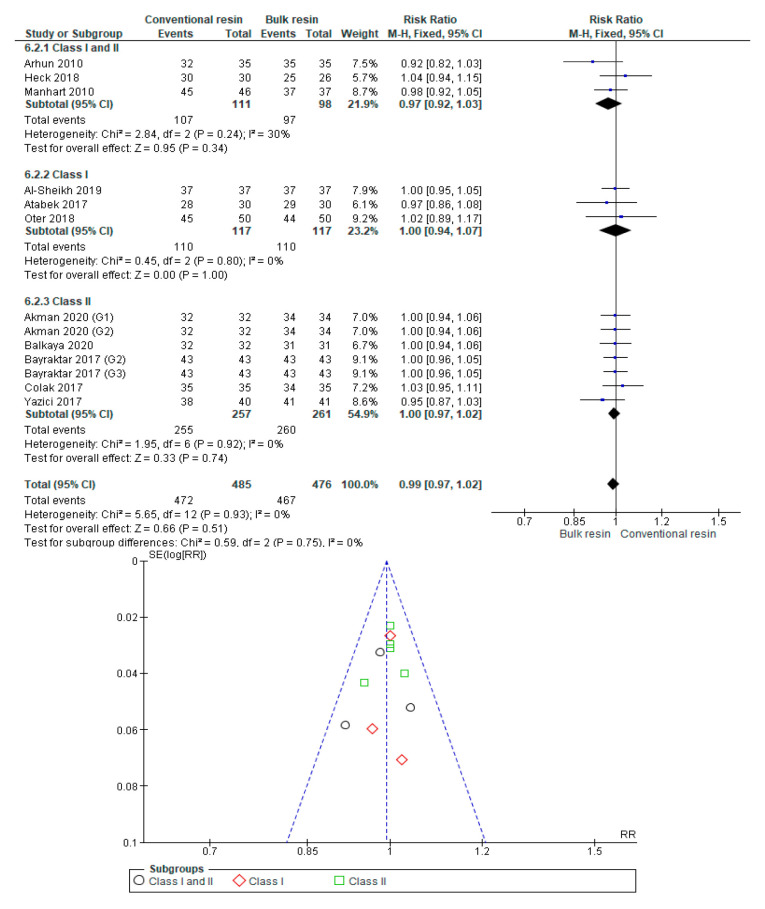
Adequate Color Stability and Translucency: Conventional Resin versus Bulk Resin According to Type of Restoration.

**Figure A28:**
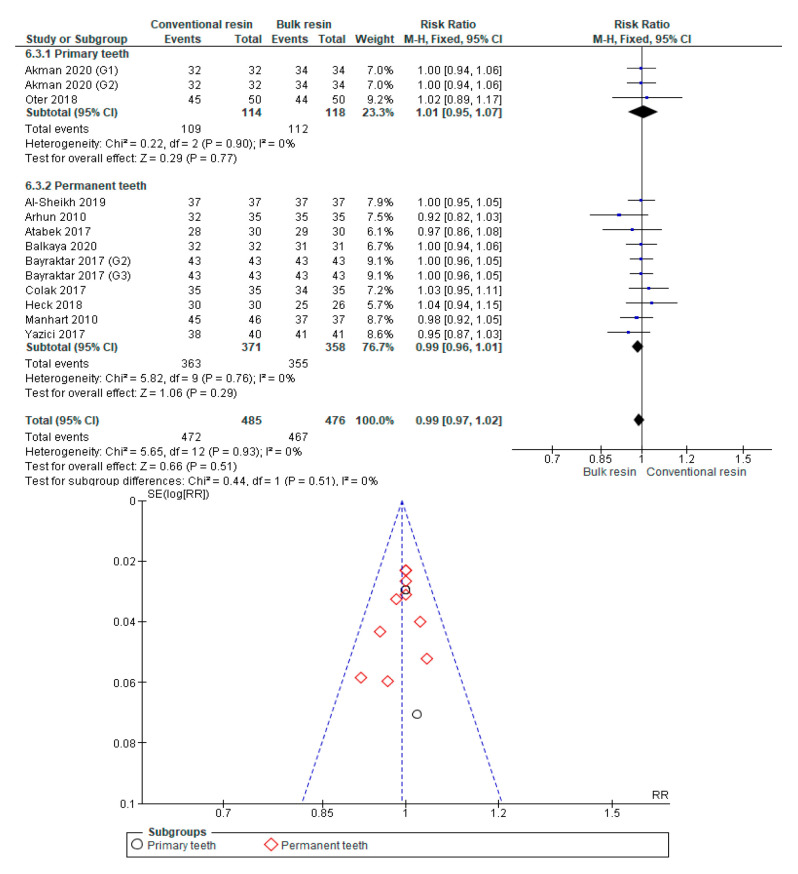
Adequate Color Stability and Translucency: Conventional Resin versus Bulk Resin According to Type of Teething.

**Figure A29:**
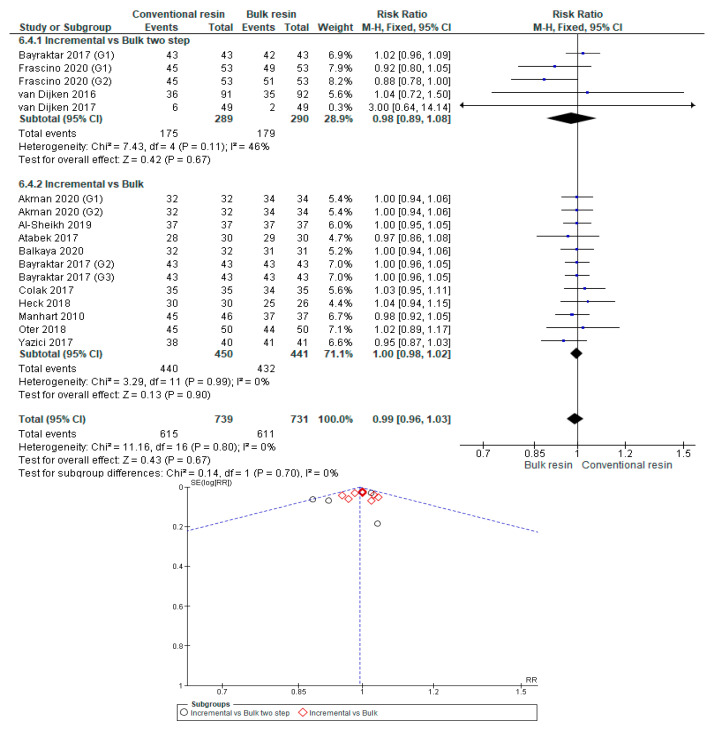
Adequate Color Stability and Translucency: Conventional Resin versus Bulk Resin According to the Technique Used.

**Figure A30:**
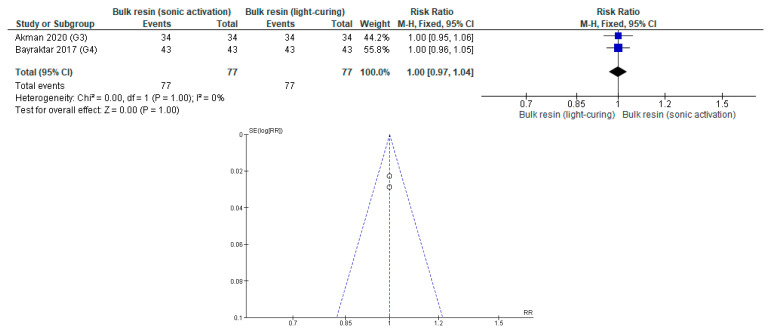
Adequate Color Stability and Translucency: Bulk Resin (with Sonic Activation) versus Bulk Resin (Two-Step Technique).

**Figure A31:**
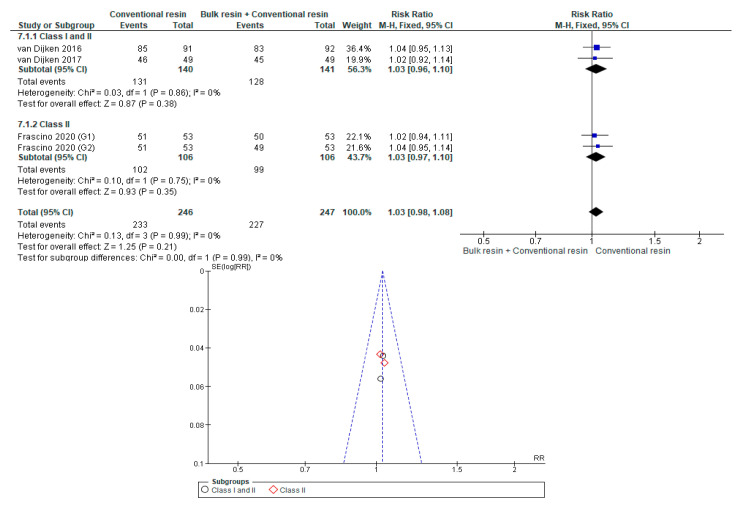
Proper Surface Texture: Conventional Resin versus Bulk Resin Covered with Conventional Resin According to Type of Restoration.

**Figure A32:**
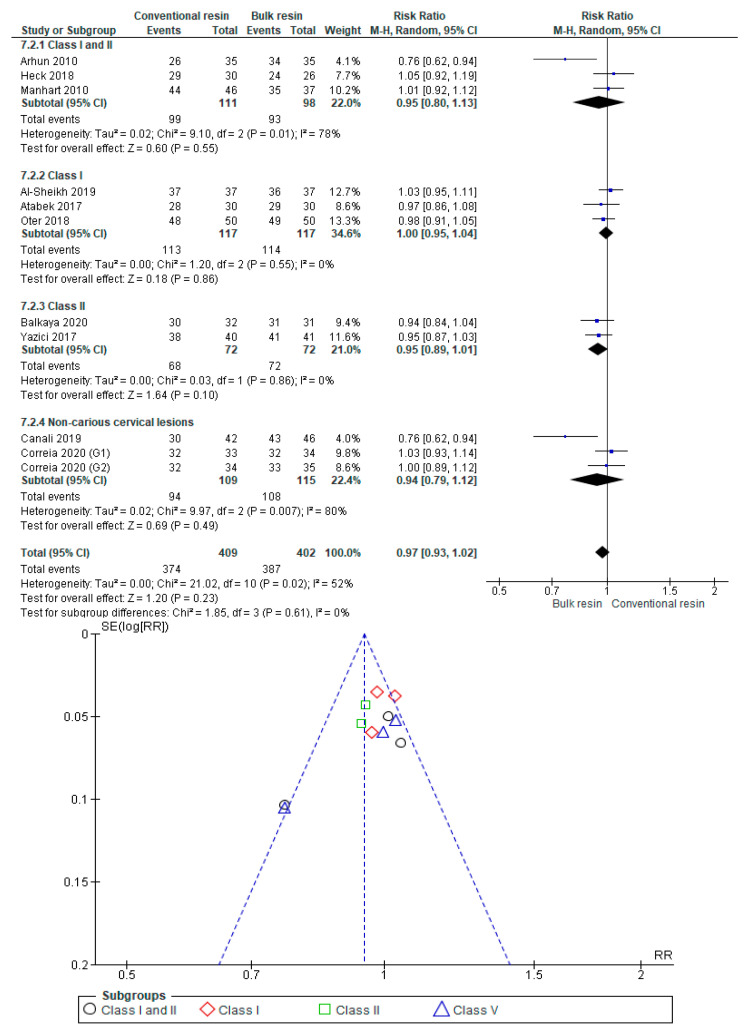
Proper Surface Texture: Conventional Resin versus Bulk Resin According to Type of Restoration.

**Figure A33:**
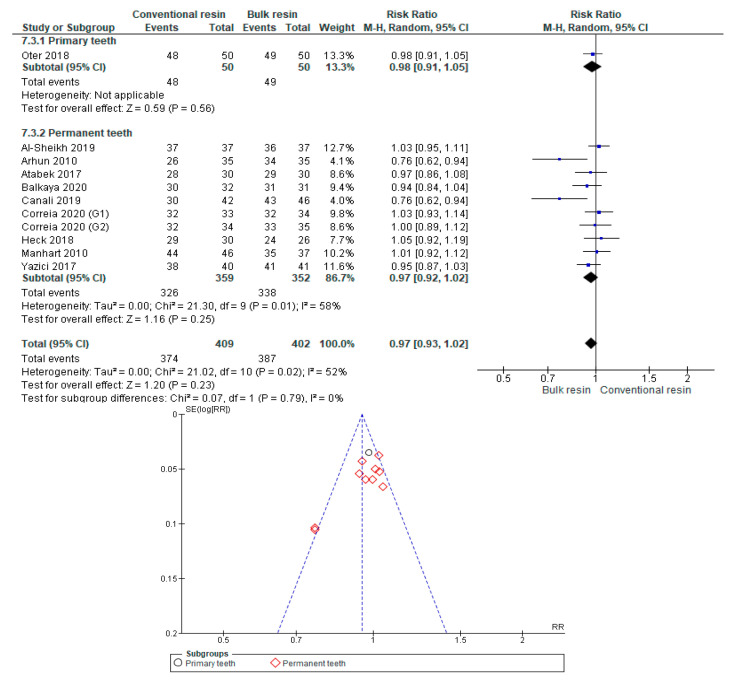
Proper Surface Texture: Conventional Resin versus Bulk Resin According to Type of Teething.

**Figure A34:**
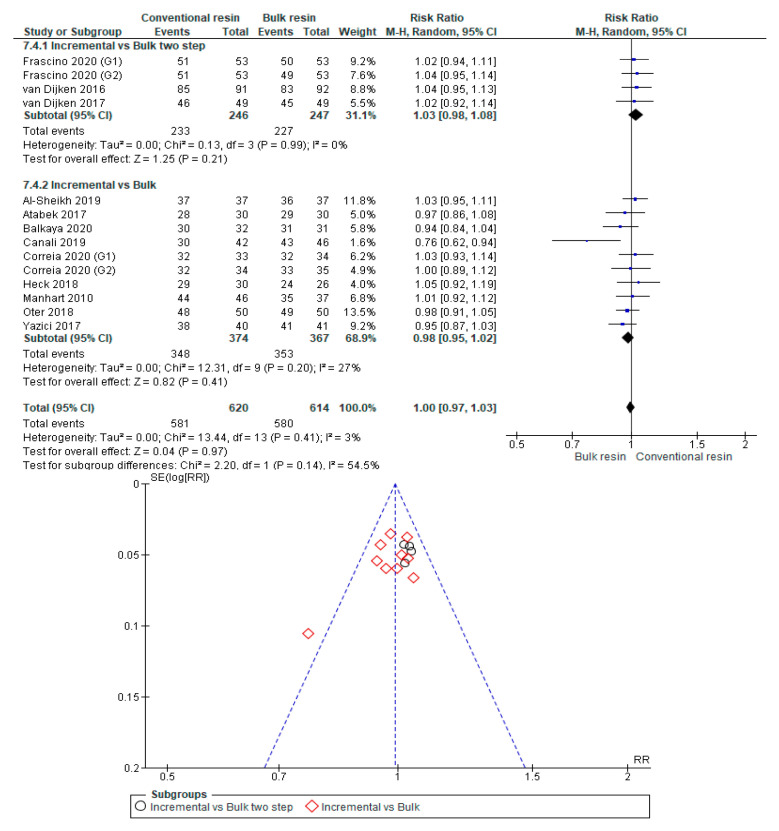
Proper Surface Texture: Conventional Resin versus Bulk Resin According to the Technique Used.

**Figure A35:**
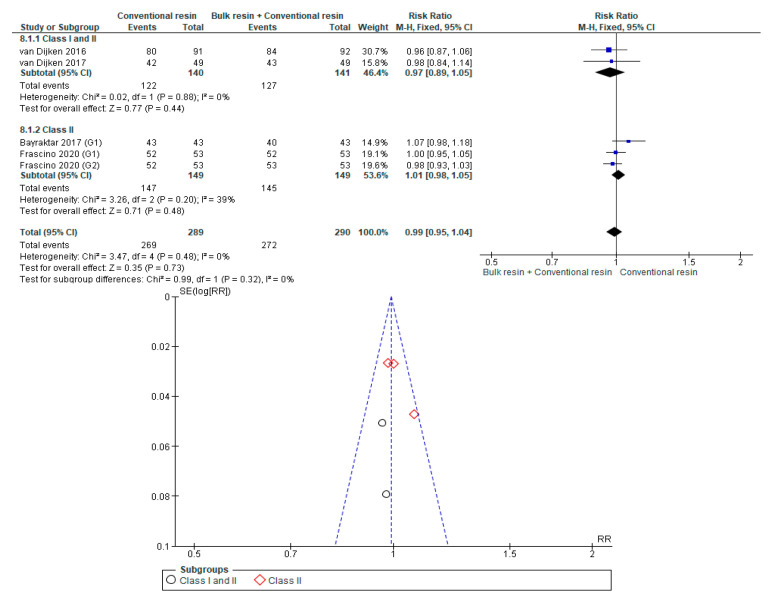
Proper Anatomical Form: Conventional Resin versus Bulk Resin Covered with Conventional Resin According to Type of Restoration.

**Figure A36:**
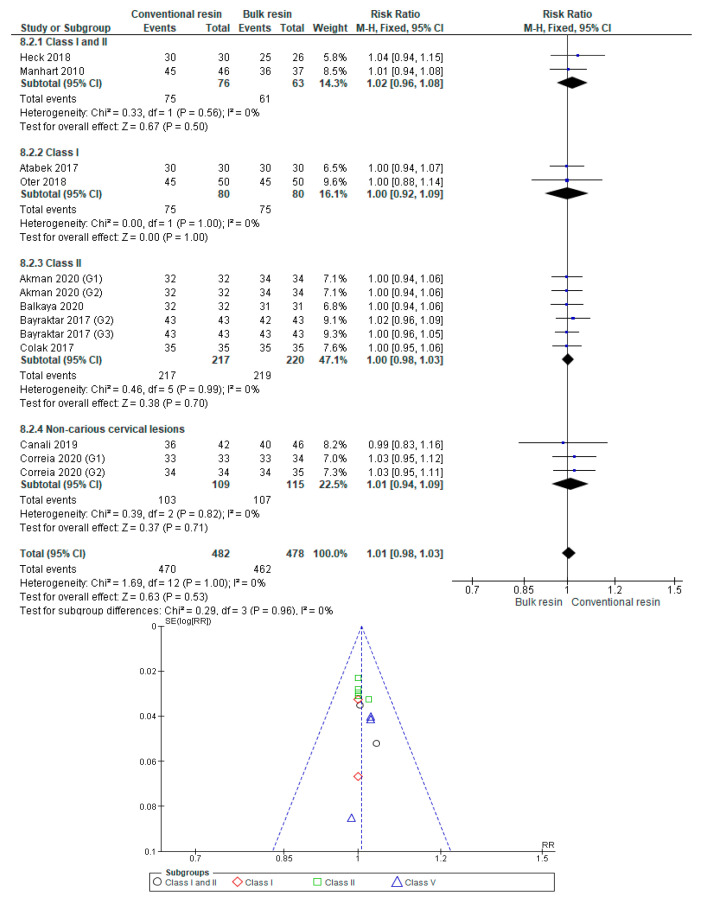
Proper Anatomical Form: Conventional Resin versus Bulk Resin According to Type of Restoration.

**Figure A37:**
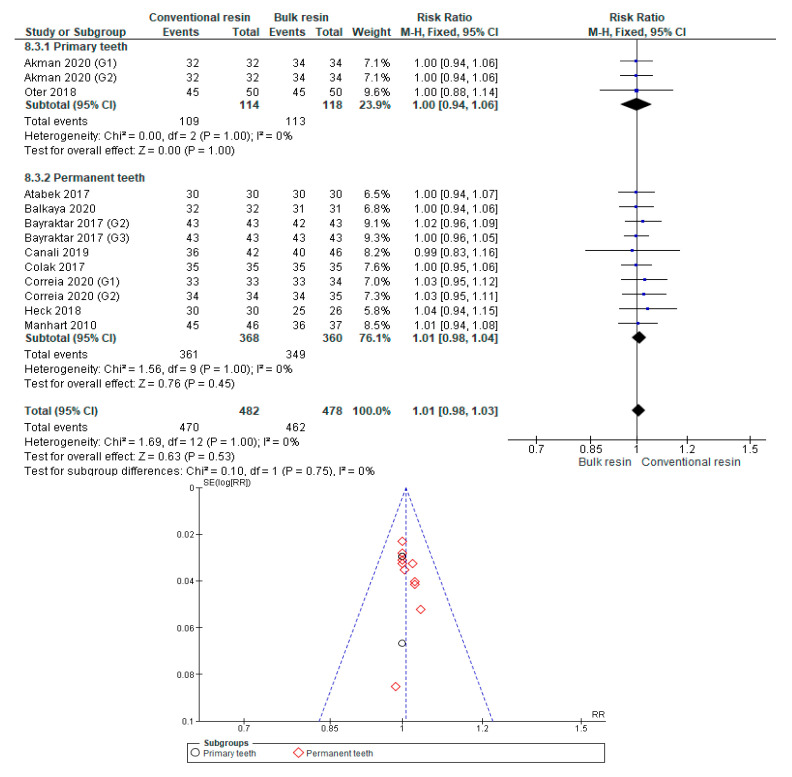
Proper Anatomical Form: Conventional Resin versus Bulk Resin According to Type of Teething.

**Figure A38:**
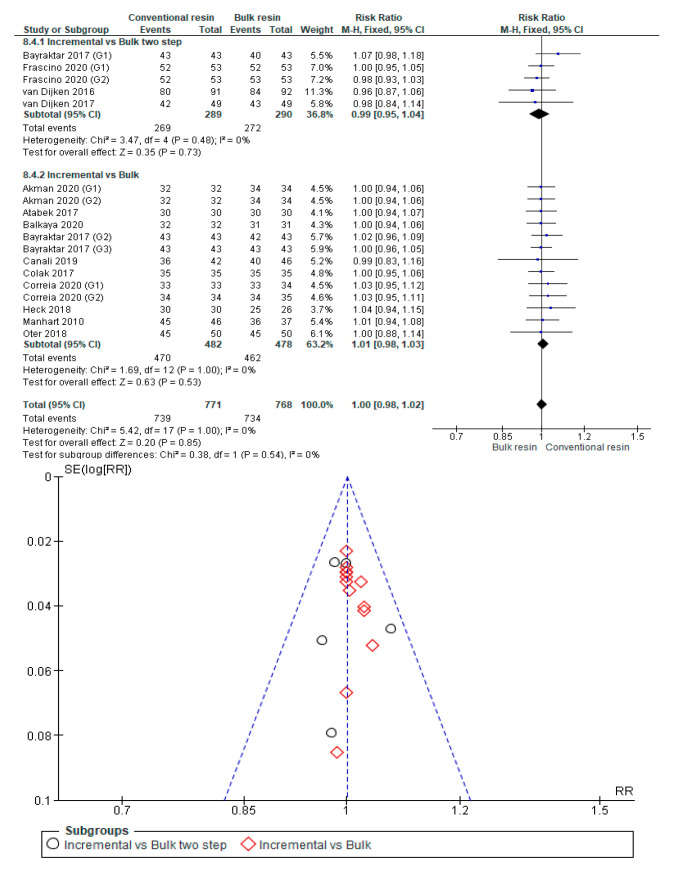
Proper Anatomical Form: Conventional Resin versus Bulk Resin According to the Technique Used.

**Figure A39:**
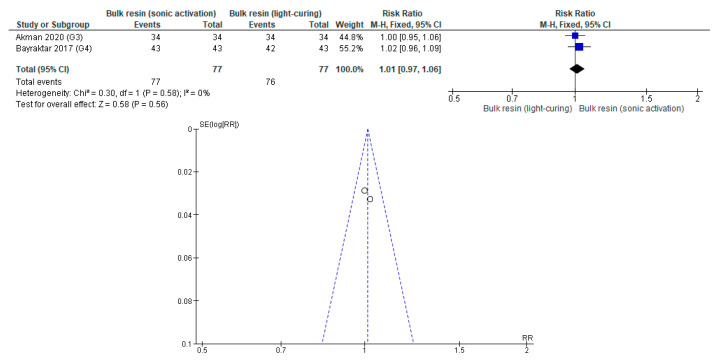
Proper Anatomical Form: Bulk Resin (with Sonic Activation) versus Bulk Resin (Two-Step Technique).

**Figure A40:**
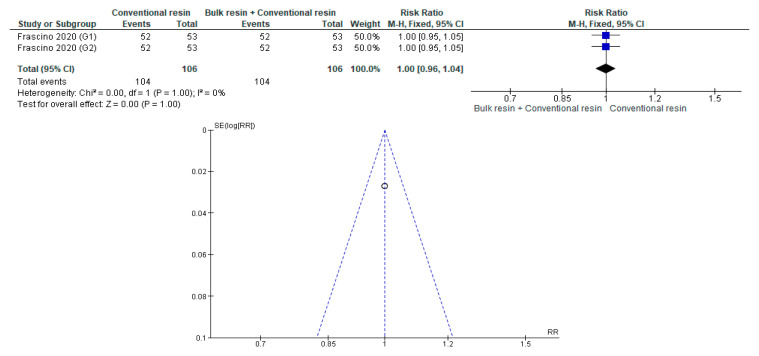
Adequate Tooth Integrity: Conventional Resin versus Bulk Resin Covered with Conventional Resin.

**Figure A41:**
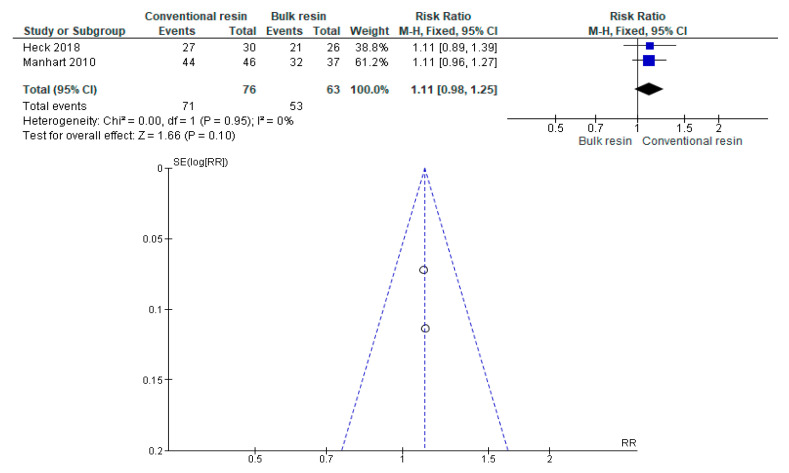
Adequate Tooth Integrity: Conventional Resin versus Bulk Resin.

**Figure A42:**
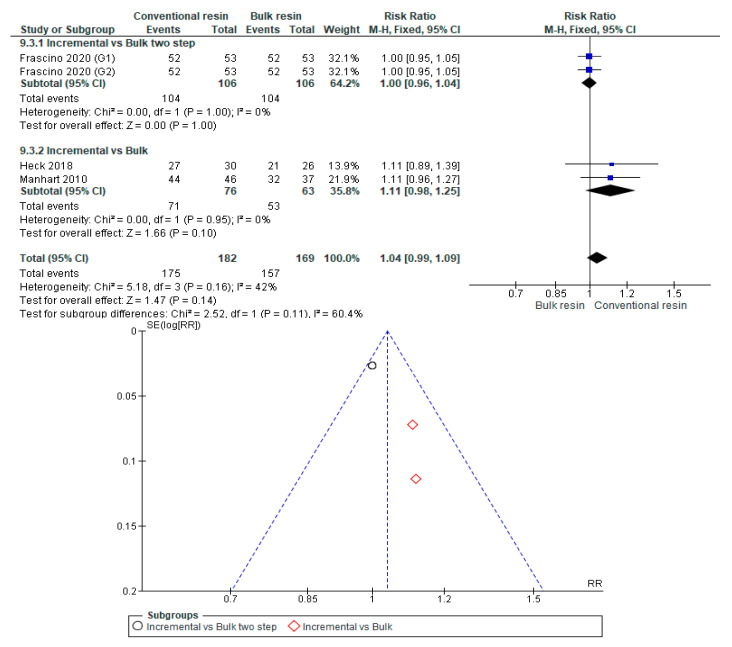
Adequate Tooth Integrity: Conventional Resin versus Bulk Resin According to the Technique Used.

**Figure A43:**
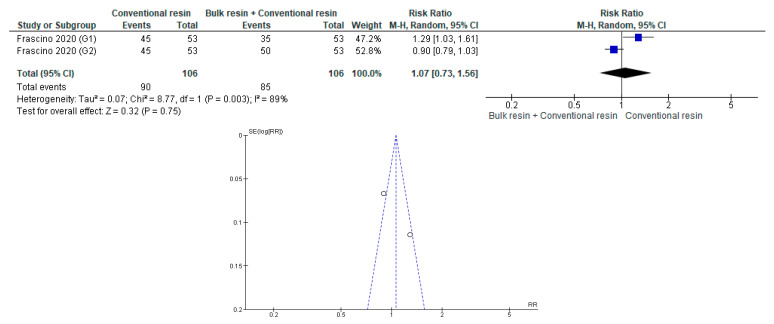
Adequate Restoration Integrity: Conventional Resin versus Bulk Resin Covered with Conventional Resin.

**Figure A44:**
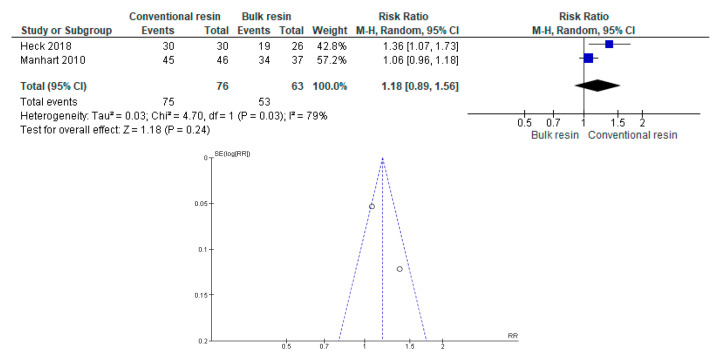
Adequate Restoration Integrity: Conventional Resin versus Bulk Resin.

**Figure A45:**
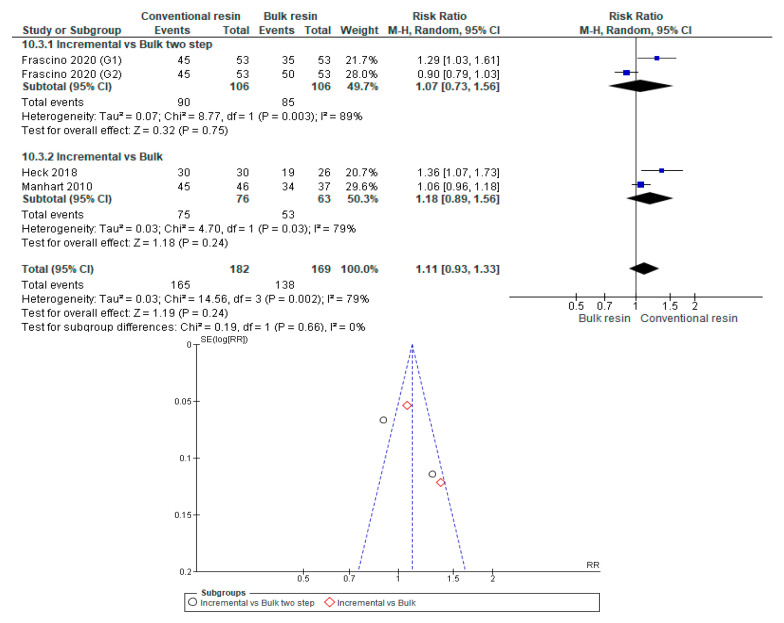
Adequate Restoration Integrity: Conventional Resin versus Bulk Resin According to the Technique Used.

**Figure A46:**
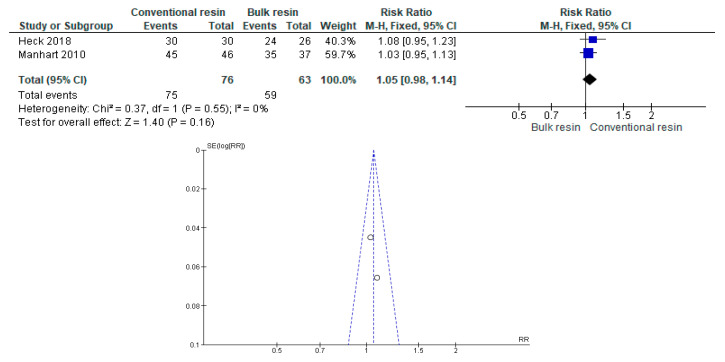
Proper Occlusion: Conventional Resin versus Bulk Resin.

## Appendix A

The meta-analyses of the data extracted from the included studies is presented. The data involved the following 11 clinical parameters: absence of fractures, absence of discoloration or marginal staining, adequate marginal adaptation, absence of post-operative sensitivity, absence of secondary caries, adequate color stability and translucency, proper surface texture, proper anatomical form, adequate tooth integrity (no wear), adequate restoration integrity, and proper occlusion. The results are presented as forest plots and funnel plots; where: CI = confidence interval; df = degrees of freedom; M-H = Mantel-Haenszel; I^2^ = Higgins I^2^ test; Chi^2^ = Chi square test.
